# Disease burden and long-term trends of urinary tract infections: A worldwide report

**DOI:** 10.3389/fpubh.2022.888205

**Published:** 2022-07-27

**Authors:** Xiaorong Yang, Hui Chen, Yue Zheng, Sifeng Qu, Hao Wang, Fan Yi

**Affiliations:** ^1^Clinical Epidemiology Unit, Qilu Hospital, Shandong University, Jinan, China; ^2^Department of Emergency Medicine, Shandong Provincial Clinical Research Center for Emergency and Critical Care Medicine, Key Laboratory of Emergency and Critical Care Medicine of Shandong Province, Chest Pain Center, Institute of Emergency and Critical Care Medicine of Shandong University, Qilu Hospital, Shandong University, Jinan, China; ^3^Department of Urology, Qilu Hospital, Shandong University, Jinan, China; ^4^Department of Critical Care Medicine, Qilu Hospital, Shandong University, Jinan, China; ^5^The Key Laboratory of Infection and Immunity of Shandong Province, Department of Pharmacology, School of Basic Medical Sciences, Shandong University, Jinan, China

**Keywords:** urinary tract infection, global burden, temporal trend, disparity, systematic analysis

## Abstract

**Background:**

Urinary tract infections (UTIs) are one of the most common infections worldwide, but little is known about their global scale and long-term trends. We aimed to estimate the spatiotemporal patterns of UTIs' burden along with its attributable risk factors at a global level, as well as the variations of the burdens according to socio-demographic status, regions, nations, sexes, and ages, which may be helpful in guiding targeted prevention and treatment programs.

**Methods:**

Data from the Global Burden of Disease Study 2019 were analyzed to depict the incidence, mortality, and disability-adjusted life years (DALYs) of UTIs in 204 countries and territories from 1990 to 2019 by socio-demographic status, nations, region, sex, and age.

**Results:**

Globally, 404.61 million cases, 236,790 deaths, and 520,200 DALYs were estimated in 2019. In particular, 2.4 times growth in deaths from 1990 to 2019 was observed, along with an increasing age-standardized mortality rate (ASMR) from 2.77/100,000 to 3.13/100,000. Age-standardized incidence rate (ASIR) was consistently pronounced in regions with higher socio-demographic index (SDI), which presented remarkable upward trends in ASMR and age-standardized DALY rate (ASDR). In contrast, countries with a low SDI or high baseline burden achieved a notable decline in burden rates over the past three decades. Although the ASIR was 3.6-fold higher in females than males, there was no sex-based difference in ASMR and ASDR. The burden rate typically increased with age, and the annual increasing trend was more obvious for people over 60 years, especially in higher SDI regions.

**Conclusions:**

The burden of UTIs showed variations according to socio-demographic status, nation, region, sex, and age in the last three decades. The overall increasing burden intimates that proper prevention and treatment efforts should be strengthened, especially in high-income regions and aging societies.

## Introduction

Urinary tract infections (UTIs) are one of the most common infections worldwide. UTIs are associated with a decrease in the quality of life of patients and a significant clinical and economic burden (1). In both community and hospital settings, UTIs pose a threat to public health. They are the most common outpatient infections (2) and at least half of adult women will have more than one UTI in their lifetime (3). In healthcare settings, the percentage of patients diagnosed with healthcare-associated UTIs is as high as 9.4% (2).

UTIs are heterogeneous with regard to their etiology, clinical manifestations, and disease course, which range from simple (e.g., urethritis and cystitis) to severe (e.g., pyelonephritis, bacteremia, and septic shock) (4). Furthermore, the pathogenic microorganisms of UTI are various, with significant changes by years and differences by countries or regions (5). A wide range of virulence factors and multi-drug resistant pathogenic strains are involved in the pathogenicity and resistance of the uropathogenic agents, making it more difficult to manage these complicated infections (6–8). Despite the advances in their diagnosis and management, UTIs are still related to high incidence and mortality rates (9), especially in healthcare settings. For example, in hospitalized patients, UTIs are associated with an attributed mortality rate of 2.3% and an estimated annual cost of $340 to $450 million in the United States (10, 11).

There are limited data on the global scale and long-term trends of UTIs. Comprehensive national- and regional-level information about the UTI burden is important for policymakers with regard to allotting the finite resources available and establishing effective public health policies. The Global Burden of Disease (GBD) 2019 study is a systematic global epidemiological study that quantified the incidence, mortality, disability, and 87 risk factors for 369 diseases by sex, age, location, and year (12, 13). Our research summarized the incidence, mortality, disability-adjusted life years (DALYs), and the long-term trends of UTIs according to age and sex in 204 countries and territories from 1990 to 2019, based on the data of the Global Burden of Disease (GBD) 2019 study.

The present research produces the latest long-term global estimate of the burden of UTIs and provides a comprehensive picture of the specific characteristics of this disease in different countries and regions, socio-demographic status, age groups, and sexes. The correlations between different parameters of UTI burden are also disclosed and explore the potential attribute factors for the changing trends. Considering most UTIs are preventable and cured well under proper health management, we believe our data will help in the formulation of targeted strategies for reducing the burden of UTIs.

## Materials and methods

### Data source

Previous research has described methods for gathering and analyzing the original data of the GBD 2019 study (12, 13). Here, we briefly introduce methods specific to UTIs estimation, and the detailed information on methods could be retrieved from Supplementary materials. The study was performed through a dry laboratory (*in-silico*) method (14). The methodology for analyzing the GBD database in the current study was in accordance with the Guidelines for Accurate and Transparent Health Estimates Reporting (GATHER) statement (15). For assessment of the GBD 2019 study, UTIs are defined as an infection of the urinary system that involves the kidneys, urethra, bladder, or ureter, accompanied by infectious manifestations (16, 17). The burden information of UTIs was collected from various vital registration data, verbal autopsy data, hospital discharges, and claims data by searching the International Classification of Diseases codes related to all kinds of UTIs (13). In GBD 2019 study, the UTIs' burden was estimated using a DisMod-MR Bayesian meta-regression model to produce estimates by age, sex, year, and country. We collected data about the worldwide burden of UTIs from 1990 to 2019 according to sex and 5-year age groups from the website of the Institute for Health Metrics and Evaluation (http://ghdx.healthdata.org/gbd-2019). Based on the socio-demographic index (SDI), which is a composite indicator of income per person, years of education, and fertility rate, the 204 countries and territories included in the GBD 2019 study were divided into five categories: low, low-middle, middle, high-middle, and high SDI regions. Furthermore, the world was divided into 21 GBD regions, including High-income North America, Western Sub-Saharan Africa, and South Asia, and so on.

### Statistical analysis

Age-standardized incidence rate (ASIR), age-standardized mortality rate (ASMR), and age-standardized DALY rate (ASDR) were used to assess differences in the burden of UTIs according to historical period, sex, and location, for eliminating differences caused by the age composition of the population (18). The 95% uncertainty intervals (UIs) for each indicator in the GBD study were estimated based on the 2.5% and 97.5% values of random 1,000 draws of the posterior distribution (13). The estimated annual percentage change (EAPC) was calculated to depict the secular tendency in various burden rates of UTI based on a regression model, in which we fitted the natural logarithm of burden rate with the calendar year, as follows: ln (burden rate) = α + β^*^ calendar year + ε (19–22). The EAPC and its 95% confidence interval (CI) were estimated based on the formula 100 × (exp (β)-1). The age-standardized indicator was considered to exhibit an increasing trend when the EAPCs and the lower boundary of the 95%CI were positive; conversely, it was considered to exhibit a decreasing trend when the EAPCs and the upper boundary of the 95%CI were negative. Spearman rank correlation was used to estimate the correlation of the EAPCs in UTI burden with SDI in 2019 and with the baseline burden in 1990 in the 204 countries and territories under the assumption of a non-normal distribution (23, 24). The ASRs of UTIs in 1990 were considered to represent the baseline reservoir of the disease, while the SDI in 2019 was considered to represent the availability and ability of medical care and public health in every country (23, 24). Statistical analyses were conducted with R (version 4.0.3). A flow chart of this study was made according to a previous recommendation to show the procedures (25), as shown in Supplementary materials. Two-sided *P* < 0.05 were considered to indicate statistical significance.

## Results

### Disease burden and temporal trend in UTIs at the global level

**Figure 1:**
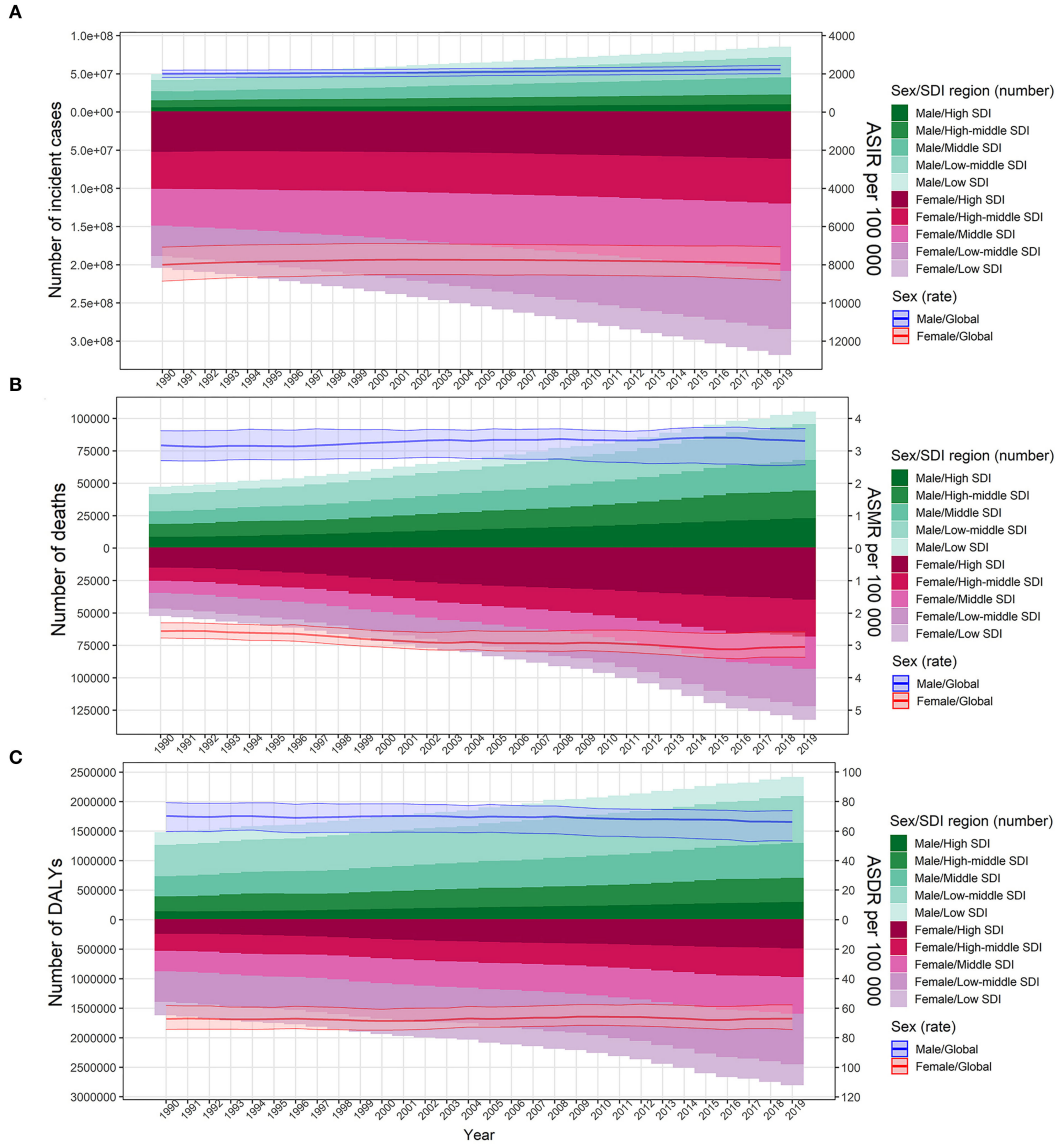
Number of cases and age-standardized rates of urinary tract infection burden according to sex for the period 1990 to 2019. The bar plot presented the absolute burden of urinary tract infection, and the line plot presented the age-standardized burden rate of urinary tract infection. **(A)** Incidence of urinary tract infection. **(B)** Mortality associated with urinary tract infection. **(C)** DALYs associated with urinary tract infection. DALYs, disability-adjusted life years; SDI, socio-demographic index; ASIR, age-standardized incidence rate; ASMR, age-standardized mortality rate; ASDR, age-standardized DALY rate.

The absolute number of cases of UTIs increased by 60.40% from 252.25 million (95%UI: 223.31–279.3) in 1990 to 404.61 million (95%UI: 359.43–446.55) in 2019. However, ASIR was relatively stable from 49.9 (95%UI: 44.34–54.99) to 50.76 (95%UI: 45.17–55.94) per 1,000 person-years over the 30-year study period, with an EAPC of 0.08 (95%CI: 0.04–0.12) (Supplementary Table S1; Figure 1A). The global deaths due to UTIs were 236,790 (95%UI: 198,430–259,030) in 2019, which increased by 140.18% from 98,590 (95%UI: 89,030–106,320) deaths in 1990. ASMR increased by 13.00% from 2.77 (95%UI: 2.51–3.02) to 3.13 (95%UI: 2.61–3.43) per 100,000 person-years over the study period, with an EAPC of 0.55 (95%CI: 0.47–0.62; Table 1, Figure 1B). Globally, the number of DALYs caused by UTIs was 520,200 (95%UI: 445,400–570,500) in 2019, which had a 68.90% increase compared with 308,000 (95%UI: 2,652,000–338,200) in 1990. However, ASDR was relatively stable from 67.73 (95%UI: 59.96–73.45) in 1990 to 66.17 (95%UI: 56.56–72.5) per 100,000 person-years in 2019, with an EAPC of −0.08 (95%CI: −0.11 to −0.04) (Supplementary Table S2; Figure 1C).

**Table 1:** Deaths and age-standardized mortality rate per 100,000 people for urinary tract infections in 1990 and 2019, and its estimated annual percentage change from 1990 to 2019.

**Characteristics**	**1990**		**2019**		**EAPC of ASMR (95%CI)**
	**ASMR/1,000 (95%UI)**	Deaths^*^10^6^ (95%UI)	**ASMR/1,000 (95%UI)**	Deaths^*^10^6^ (95%UI)	**from 1990 to 2019**
Global	2.77 (2.51, 3.02)	98.59 (89.03, 106.32)	3.13 (2.61, 3.43)	236.79 (198.43, 259.03)	0.55 (0.47, 0.62)
Male	3.14 (2.67, 3.59)	46.79 (39.61, 52.73)	3.27 (2.54, 3.65)	104.88 (82.41, 117.43)	0.28 (0.22, 0.34)
Female	2.56 (2.31, 2.77)	51.80 (46.42, 56.17)	3.05 (2.58, 3.37)	131.91 (111.61, 145.72)	0.70 (0.61, 0.78)
SDI region					
High SDI	2.42 (2.15, 2.85)	24.84 (22.14, 29.29)	2.77 (2.28, 3.02)	63.74 (51.45, 69.97)	0.60 (0.39, 0.82)
High-middle SDI	2.08 (1.84, 2.21)	20.00 (17.46, 21.10)	2.62 (2.07, 2.86)	49.93 (39.60, 54.48)	1.14 (1.00, 1.27)
Middle SDI	2.09 (1.84, 2.31)	19.00 (16.71, 20.76)	2.33 (2.00, 2.64)	48.41 (42.11, 54.11)	0.47 (0.42, 0.52)
Low-middle SDI	4.19 (3.37, 4.80)	25.43 (19.83, 29.23)	4.63 (3.58, 5.36)	56.55 (43.24, 65.60)	0.31 (0.22, 0.40)
Low SDI	3.76 (3.10, 4.52)	9.28 (7.85, 11.08)	3.72 (3.06, 4.47)	18.04 (15.06, 21.59)	−0.11 (−0.17, −0.04)
GBD Region					
High-income Asia Pacific	1.37 (1.16, 2.09)	2.21 (1.89, 3.42)	1.56 (1.18, 1.78)	9.97 (7.27, 11.47)	1.01 (0.69, 1.33)
High-income North America	3.38 (2.89, 3.62)	12.56 (10.76, 13.43)	3.44 (2.96, 3.85)	24.47 (20.94, 27.46)	−0.08 (−0.37, 0.20)
Western Europe	1.75 (1.53, 2.34)	10.11 (8.91, 13.62)	2.85 (2.07, 3.16)	33.75 (24.15, 37.55)	2.19 (1.81, 2.56)
Australasia	1.82 (1.61, 2.08)	0.39 (0.34, 0.44)	2.26 (1.83, 2.56)	1.33 (1.06, 1.51)	0.86 (0.50, 1.21)
Southern Latin America	2.13 (1.88, 2.98)	0.86 (0.77, 1.19)	7.71 (4.76, 8.68)	6.66 (4.10, 7.50)	4.92 (4.26, 5.59)
Andean Latin America	2.88 (2.48, 3.84)	0.58 (0.51, 0.76)	4.01 (2.70, 5.13)	2.13 (1.44, 2.73)	1.59 (1.08, 2.10)
Tropical Latin America	4.51 (4.03, 5.83)	3.54 (3.23, 4.57)	9.38 (5.71, 10.45)	20.99 (12.87, 23.37)	3.50 (3.14, 3.87)
Central Latin America	2.99 (2.69, 3.55)	2.34 (2.13, 2.79)	4.34 (3.49, 5.01)	9.79 (7.89, 11.31)	1.98 (1.66, 2.30)
Caribbean	1.53 (1.33, 2.05)	0.38 (0.34, 0.50)	2.99 (2.21, 3.55)	1.54 (1.14, 1.83)	2.45 (2.06, 2.85)
Eastern Europe	3.13 (2.28, 3.40)	8.45 (6.20, 9.19)	3.17 (2.72, 3.61)	10.64 (9.12, 12.08)	−0.43 (−0.79, −0.07)
Central Europe	2.97 (2.20, 3.19)	4.11 (3.02, 4.42)	1.75 (1.50, 2.09)	3.86 (3.32, 4.60)	−1.44 (−2.17, −0.70)
Central Asia	2.32 (2.08, 2.47)	1.20 (1.08, 1.28)	4.55 (3.71, 5.13)	3.35 (2.79, 3.78)	2.56 (2.18, 2.95)
North Africa and Middle East	1.06 (0.82, 1.88)	1.54 (1.26, 2.40)	1.13 (0.96, 1.46)	3.75 (3.23, 4.79)	0.41 (0.11, 0.71)
South Asia	5.41 (4.34, 6.20)	29.9 (23.34, 34.48)	5.36 (4.12, 6.28)	66.77 (51.04, 78.78)	−0.16 (−0.30, −0.03)
Southeast Asia	3.21 (2.35, 3.75)	7.93 (5.75, 8.99)	3.17 (2.40, 4.11)	16.87 (12.46, 21.21)	−0.19 (−0.30, −0.08)
East Asia	0.92 (0.74, 1.08)	6.59 (5.25, 7.64)	0.66 (0.56, 0.80)	10.61 (8.96, 13.13)	−1.15 (−1.32, −0.98)
Oceania	3.17 (2.41, 4.82)	0.08 (0.06, 0.10)	2.84 (2.28, 3.66)	0.17 (0.13, 0.21)	−0.43 (−0.48, −0.39)
Western Sub-Saharan Africa	2.69 (2.16, 3.40)	2.53 (2.05, 3.21)	2.17 (1.67, 3.19)	4.19 (3.19, 6.09)	−0.93 (−1.02, −0.83)
Eastern Sub-Saharan Africa	3.05 (1.87, 4.80)	2.34 (1.54, 3.57)	2.69 (1.47, 4.38)	4.08 (2.30, 6.54)	−0.51 (−0.56, −0.45)
Central Sub-Saharan Africa	2.85 (1.80, 4.56)	0.59 (0.40, 0.91)	2.59 (1.45, 4.31)	1.21 (0.69, 2.00)	−0.44 (−0.51, −0.36)
Southern Sub-Saharan Africa	1.23 (0.85, 1.46)	0.34 (0.23, 0.40)	1.26 (1.04, 1.59)	0.64 (0.52, 0.80)	−0.08 (−0.69, 0.53)

*ASMR, age-standardized mortality rate; No., number; UI, uncertainty interval; EAPC, estimated annual percentage change; CI, confidential interval*.

### Variation in UTI burden according to different socio-demographic index

The ASIR of UTIs was the highest in high SDI regions across all years: 68.68/1,000 in 1990 and 64.24/1,000 in 2019 (Supplementary Table S1). In contrast, middle SDI regions had the lowest ASIR: 37.31/1,000 in 1990 and 43.92/1,000 in 2019. During the study period from 1990 to 2019, a significant increase in ASIR was observed in the middle SDI regions (EAPC = 0.63) (Supplementary Table S1).

In 2019, the low-middle SDI regions had the highest ASMR of 4.63/100,000, while the low SDI regions ranked second with an ASMR of 3.72/100,000. The middle SDI regions had the lowest ASMR of 2.33/100,000 in 2019 (Table 1). The highest increase in ASMR was observed in the high-middle SDI regions (EAPC = 1.14), and the high SDI regions had the second-highest increase in ASMR (EAPC = 0.60). In contrast, the low SDI regions showed a decrease in ASMR from 1990 to 2019 (EAPC = −0.11).

The highest ASDR in 2019 was observed in low-middle SDI regions at 110.74/100,000. With the exception of the high-SDI regions (EAPC = 0.34), the ASDR in the other four SDI regions decreased over the study period, especially the ASDR of the low SDI regions (EAPC = −0.55) (Supplementary Table S2).

### Variation in UTI burden at the regional and national level

With regard to the 21 GBD regions, Andean Latin America, Tropical Latin America, and Australasia had the highest ASIR in 2019, with their ASIRs ranging from 131.64 to 96.63 per 1,000 person-years (Supplementary Table S1). The three GBD regions with the lowest ASIR in 2019 were East Asia, Oceania, and Southeast Asia, with their ASIRs ranging from 12.31 to 23.63 per 1,000 person-years (Supplementary Table S1). The ASIR of UTIs in Central Latin America, Andean Latin America, and South Asia showed an increase from 1990 to 2019, with the EAPC ranging from 0.48 to 0.33. In contrast, the ASIR of UTIs in the other GBD regions showed a stable or downward trend from 1990 to 2019 (Supplementary Table S1).

In contrast to the largely stable trend observed for ASIR, ASMR showed an obvious upward trend in most GBD regions from 1990 to 2019, including Southern Latin America, Tropical Latin America, Central Asia, the Caribbean, Western Europe, Central Latin America, and Andean Latin America. In particular, Tropical Latin America, Southern Latin America, and South Asia had the highest ASMR in 2019 (more than 5.0/100,000). The ASMR of Tropical Latin America and Southern Latin America increased significantly and was the highest over the 30-year period from 1990 to 2019 (EAPC > 3.5, Table 1). The lowest ASMR was observed in East Asia (0.29), which was followed by North Africa, the Middle East, and Southern Sub-Saharan Africa. Thus, the data show that the distribution pattern of the ASDR of UTIs in the GBD regions was similar to the overall pattern of ASMR (Supplementary Table S2).

**Figure 2:**
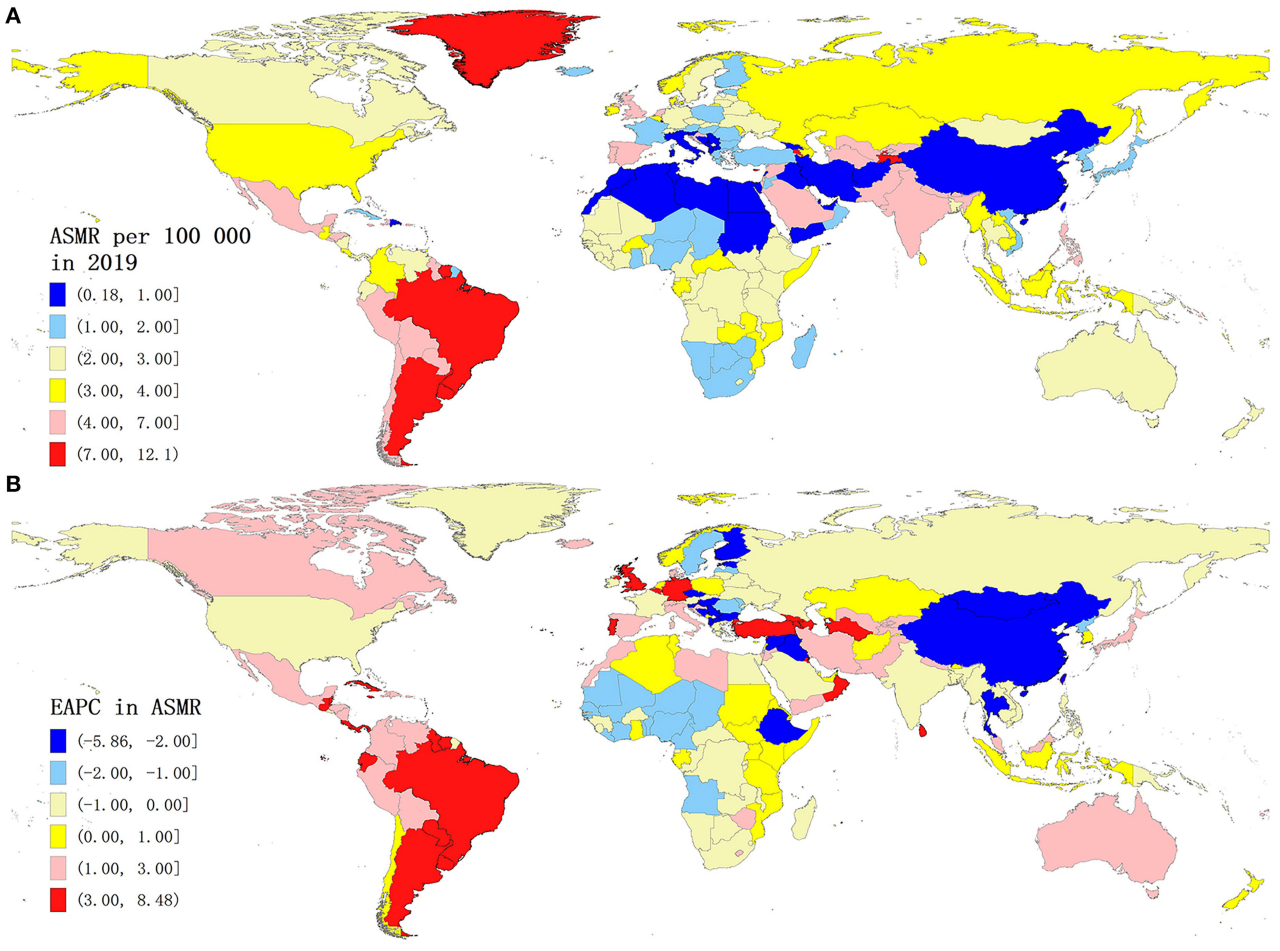
Global mortality of urinary tract infection for both sexes across 204 countries and territories. **(A)** ASMR of urinary tract infection in 2019. **(B)** EAPC in the ASMR of urinary tract infection from 1990 to 2019. ASMR, age-standardized mortality rate; EAPC, estimated annual percentage change.

In 2019, Ecuador had the highest ASIR (15.54/1,000) and North Korea had the lowest ASIR (1.21/1,000), which presented about 13 times geographic disparity across the world. Apart from Ecuador, eight other countries and territories had an ASIR above 9.5/1,000, namely, Puerto Rico, Bermuda, New Zealand, Norway, Bolivia, Peru, Brazil, and Paraguay (Supplementary Figure S1A; Supplementary Table S3). On the contrary, nine countries and territories, namely, China, Taiwan of China, Papua New Guinea, Vanuatu, Palau, Tuvalu, Solomon Islands, Marshall Islands, and Micronesia, had an ASIR lower than 1.9/1,000 in 2019 (Supplementary Figure S1A; Supplementary Table S3). The highest ASMR in 2019 was observed in Barbados (12.01/100,000), which was followed by Seychelles, Brunei, Armenia, Brazil, and Saint Kitts and Nevis (Figure 2A). North Macedonia had the lowest ASMR (0.19/100,000) in 2019, and it was followed by Montenegro, Cook Islands, Egypt, Albania, and China (Figure 2A). The geographical distribution of ASDR and ASMR was highly consistent in 2019 (Supplementary Figure S1B).

From 1990 to 2019, ASIR showed an increasing trend in 144 of 204 countries and territories. The largest annualized growth of ASIR was observed in Ecuador [EAPC = 1.10 (95%CI: 0.94–1.25)], and the EAPC in ASIR in three other countries and territories, namely, Croatia, Botswana, and Mexico, exceeded 0.50 (Supplementary Figure S1C). Conversely, the fastest decline in ASIR was found in Italy [EAPC in ASIR = −2.61 (95%CI: −3.12 to −2.10)], while the EAPC in ASIR in seven countries and territories (Poland, Indonesia, Malta, Qatar, Cyprus, USA, and Portugal) was < −0.3 (Supplementary Figure S1C).

The annualized percentage change in both ASMR and ASDR was highest in Armenia and Portugal [all EAPCs >7.0 and >6.0, respectively] and lowest in Finland and Bulgaria [all EAPCs < −5.0 and −6.0, respectively] from 1990 to 2019. The EAPC in ASMR exceeded 3.00 in 31 countries and territories, including Argentina, Kuwait, Uruguay, Turkmenistan, Belgium, and Mauritius (Table 1; Figure 2B). The EAPC in ASMR was < −1.5 in 22 countries and territories, including Finland, Estonia, Mongolia, Slovenia, Albania, and Syria. Overall, the annualized percentage change in ASDR followed a very similar pattern to that for ASMR (Table 1; Supplementary Figure S1D).

### Variation in UTI burden according to sex and age

**Figure 3:**
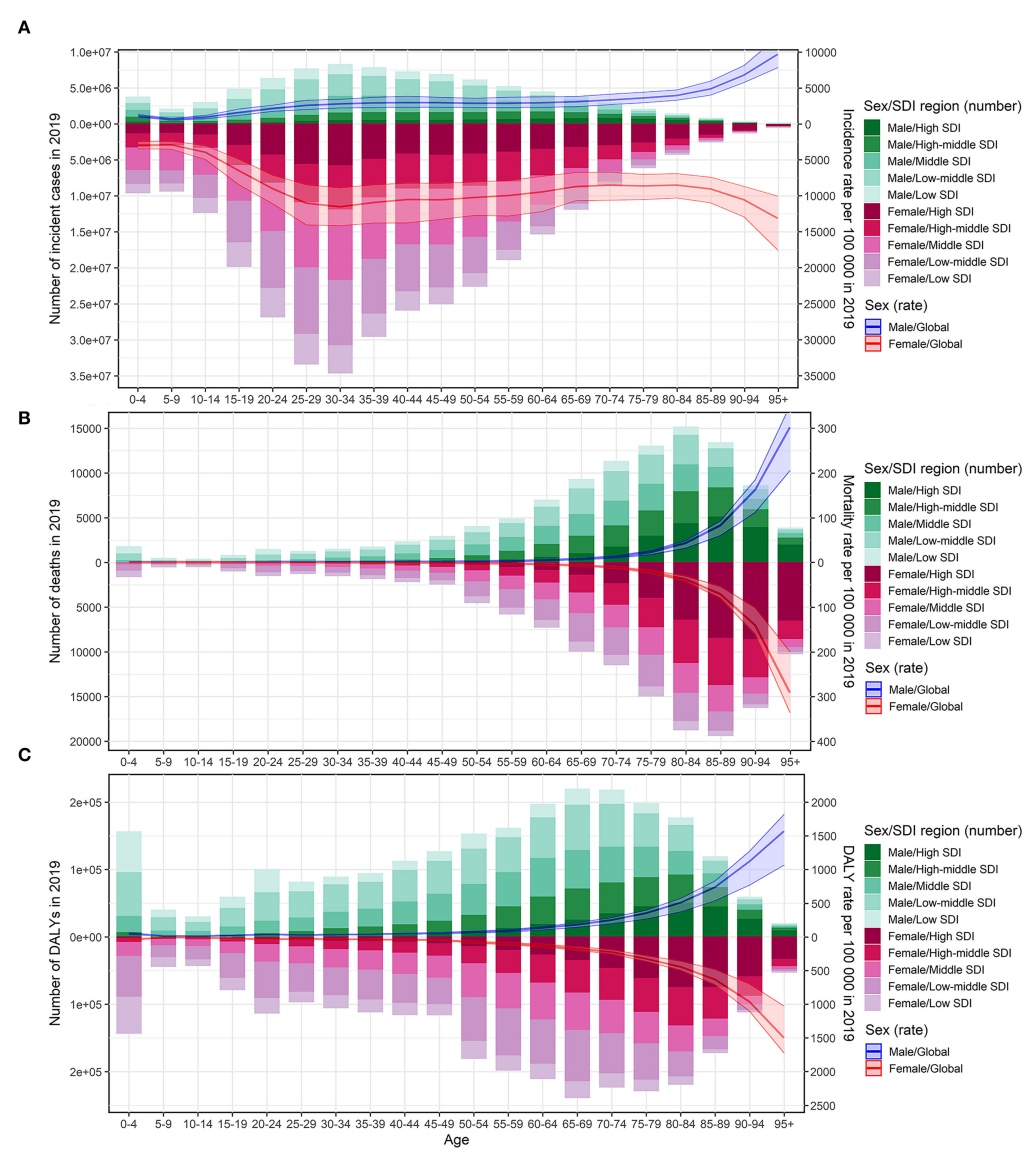
Age-specific number of cases and burden of urinary tract infection according to sex in 2019. The bar plot presented the absolute burden of urinary tract infection, and the line plot presented the age-specific burden rate of urinary tract infection. **(A)** Incidence of urinary tract infection. **(B)** Mortality associated with urinary tract infection. **(C)** DALYs associated with urinary tract infection. DALYs, disability-adjusted life years; SDI, socio-demographic index.

The ASIR of UTIs among females was 3.6 times higher than among males (79.64/1,000 vs. 22.12/1,000 in 2019) (Supplementary Table S1), but no sex-based difference was observed in ASMR and ASDR. The increase in ASMR from 1990 to 2019 was greater among females than among males at the global level (Table 1). However, the increase in ASIR and the decrease in ASDR from 1990 to 2019 were greater among males than among females (Supplementary Tables S1, S2). At a global level, in 2019, the incidence rate gradually increased with age among adolescents and reached a peak at around 35 years for both sexes (Figure 3A). Following this, it remained stable among males and slightly decreased among females, until it significantly increased in both sexes in the population aged over 80 years (Figure 3A). The mortality rate and DALYs for both sexes were low but showed a significant increase after the age of 65 to 75 years independently in both sexes (Figures 3B,C).

From 1990 to 2019, the absolute incidence of cases showed a steady rise in all SDI regions across most age groups, except for newborns, children, and young adults in the higher SDI regions (Supplementary Figure S2A). The incidence rate increased in the population over 85 years in all SDI regions, and it also increased in the population over 60 years in most SDI regions, except for the high-middle SDI regions (Supplementary Figures S2B,C). In particular, the incidence showed an increase in all age groups in the middle SDI and high-middle SDI regions. However, the incidence decreased among individuals under 60 years of age in high-SDI regions and individuals in the age group 15 to 85 years in high-middle SDI regions (Supplementary Figures S2B,C).

**Figure 4:**
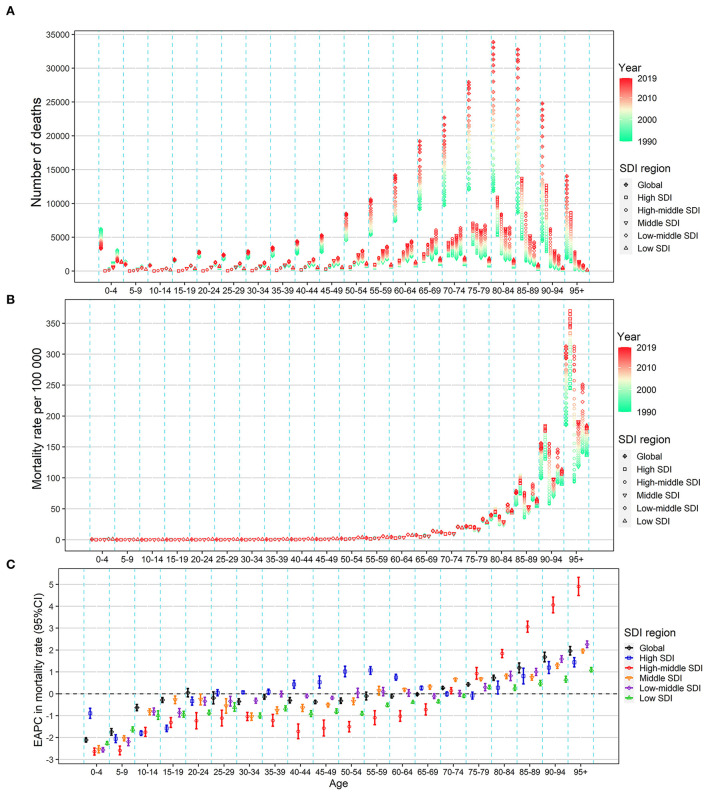
Change in the mortality of urinary tract infection across all age groups in the worldwide and five SDI regions, both sexes, from 1990 to 2019. **(A)** Number of deaths. **(B)** Age-specific mortality rate. **(C)** EAPC in the age-specific mortality rate of urinary tract infection. EAPC, estimated annual percentage change; SDI, socio-demographic index.

From 1990 to 2019, the absolute number of deaths and DALYs showed a notable rise in all SDI regions in the population aged over 15 years, especially in the elderly population over the age of 60 years (Figure 4A; Supplementary Figure S3A). Further, the mortality and DALYs showed a notable rise in all the SDI regions in the population over 80 years and showed a decrease in all the SDI regions in the population under 20 years (Figures 4B,C; Supplementary Figures S3B,C). In the age group of 20 to 75 years, the mortality and DALYs decreased in the low and high-middle SDI regions, remained stable in the middle and low-middle regions, and increased in the high SDI regions. The EAPC in ASMR and ASDR across all age groups showed a similar pattern in all SDI regions (Figures 4B,C; Supplementary Figures S3B,C).

### Correlations between parameters of different UTI burden parameters

**Figure 5:**
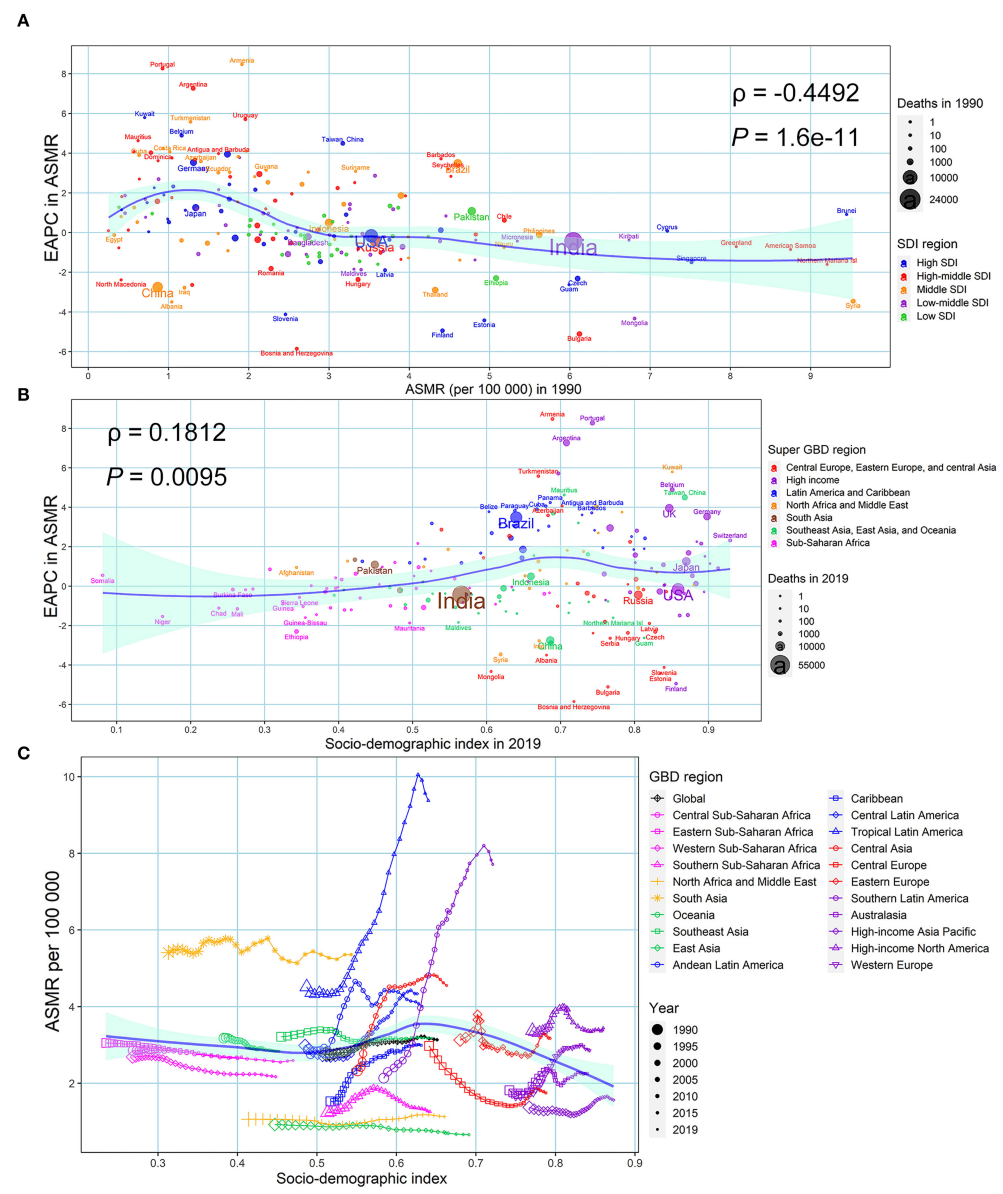
Factors associated with EAPC in ASMR associated with urinary tract infection in both sexes from 1990 to 2019. **(A)** ASMR associated with urinary tract infection in 1990 at the national and territorial level (baseline burden). **(B)** SDI associated with urinary tract infection in 2019 at the national and territorial level (development status). **(C)** Annual changing trajectory in ASMR of urinary tract infection across 21 GBD regions according to corresponding annual SDI. The blue line was fitted by LOESS. ASMR, age-standardized mortality rate; EAPC, estimated annual percentage change; SDI, socio-demographic index.

At the national and territorial level, the EAPC in ASMR and ASDR were negatively correlated with the baseline burden in 1990 (ρ = −0.449, *P* = 1.6e^−11^; ρ = −0.426, *P* =10e^−10^) (Figure 5A; Supplementary Figure S4A). A positive correlation was observed between the EAPC in ASMR and SDI (ρ = 0.181, *P* = 0.010) and between the EAPC in ASDR and SDI (ρ = 0.318, *P* = 0.049) in 2019 in the 204 countries and territories analyzed (Figure 5B; Supplementary Figure S4B). Figure 5C and Supplementary Figure S4C depict the relationship between ASMR/ASDR and SDI over time in an annual time series from 1990 to 2019 for the 21 GBD regions. The ASMR and ASDR in Tropical Latin America, Southern Latin America, Central Asia, Caribbean, Central Latin America, and West Europe presented an obvious increasing trend, especially in Tropical Latin America and Southern Latin America. In contrast, the ASMR and ASDR in Eastern Europe presented an obvious decreasing trend.

The EAPC in ASIR from 1990 to 2019 was not correlated with baseline ASIR in 1990 (ρ = −0.075, *P* = 0.286), but was negatively correlated with SDI in 2019 (ρ = −0.148, *P* = 0.034) at the national and territorial level (Supplementary Figure S5). The ASIR in most GBD regions showed a horizontal trend in most regions. However, high-income North America showed a consistently decreasing trend, while Andean Latin America and Tropical Latin America showed an increasing trend that was followed by a decrease (Supplementary Figure S5).

## Discussion

This study presents a comprehensive picture of the 30-year global burden of UTIs. Our analysis discloses that the burden of UTIs has continued to increase but showed variations according to sex, region, and age. This increase was notable in regions with higher SDI regions. The burden tended to increase with age, with the annual increasing trend being more obvious in individuals over 60 years.

During the last three decades, the ASIR was consistently pronounced in regions with higher SDI, which also presented remarkable upward trends in ASMR and ASDR. These regions have sufficient healthcare resources and high life expectancy, which are indicative of adequate available diagnosis and treatment of UTIs. Additionally, rapid and accurate diagnostic methods with higher specificity and sensitivity for detecting UTIs may be applied to detect more cases in those regions (26). However, UTI patients in higher SDI regions are often older, with decreased immunity and more complications (27–29). These may explain the higher incidence and mortality in these regions. We further explored the relationship of baseline burden in 1990 with EAPC and found that the high-burden but usually low-income countries decreased the UTI burden in terms of mortality and DALYs. In contrast, countries and territories with low baseline burdens had higher EAPCs, which illustrated that these countries afforded to increase UTI burden in the last 30 years. We also identified the key countries and territories with low baseline burden and an overall increasing trend, and these were Armenia, Portugal, Argentina, Kuwait, Uruguay, Turkmenistan, Belgium, Mauritius, Taiwan of China, Georgia, Costa Rica, Turkey, UK, and Germany. Urinary instrumentation is more common in the elderly and in developed countries. This plus the aging trend may be the explanation for the high prevalence of UTIs in this group of patients.

At the regional level, the high ASDR and ASMR of Tropical and Southern Latin America are interesting. These two GBD regions had the highest ASMR in 2019 (more than 5.0), and their ASMR showed a continuous increase over the last 30 years and presented an obvious rising trend. A possible explanation for this trend is the high prevalence of drug-resistant pathogens that cause UTIs in these regions. In fact, the SENTRY antimicrobial surveillance program demonstrated that the pathogens isolated from UTI patients in Latin America exhibited a high level of resistance to several antibiotics (30): in particular, *Pseudomonas aeruginosa* resistant to carbapenem, *Escherichia coli* resistant to ciprofloxacin, and *Klebsiella pneumoniae* with ESBL production constituted critical problems in this region. In addition, a study that analyzed urine cultures over two periods, 2005 to 2006 and 2010 to 2011, found that bacterial resistance to fluoroquinolones had been rising, and some isolates such as *Pseudomonas aeruginosa* showed a significant increase in resistance to most antibiotics (31). Further, one study showed that the resistance of uropathogens against ampicillin-sulbactam, trimethoprim/sulfamethoxazole, and cephalexin was more common than previously reported in Argentina (32). However, the specific reason deserves further exploration in the future.

UTIs are one of the most frequently diagnosed infections in older adults (33). They are responsible for 15 to 30% of all infections in this age group and also contribute to deaths and morbidities (34). In this study, the UTI burden (including incidence, mortality, and DALYs) among elderly people tended to increase with age, and the annual increasing trend was more obvious for people over 60 years of age in most SDI regions. In contrast, the mortality and DALYs decreased in all SDI regions in the population under 20 years old. The elderly are more susceptible to UTIs because of the use of urinary instrumentations and the prevalence of asymptomatic bacteriuria and residual urine. It is known that asymptomatic bacteriuria and urinary retention are more common in the geriatric population of both sexes (33, 35–37). High residual urine volume and urinary retention caused by chronic obstruction might be important causes of UTIs in older adults (38). Furthermore, UTIs are not difficult to control in younger patients but could be fatal in the elderly. Deteriorated immunologic function, exposure to nosocomial pathogens, and comorbidities increase the risk of mortality in elderly UTI patients (29). As a result, UTIs in elderly patients are more severe and more likely to result in sepsis, septic shock, and mortality (27, 28). In fact, 40 to 57% of community-acquired bacteremia cases in the geriatric population are of urinary origin (39). Furthermore, UTIs are the most common reason for antibacterial prescriptions in people living in nursing homes (33), but in 40 to 75% of cases, antimicrobial use is inappropriate (40, 41). Over-utilization of antimicrobial prescriptions increases the chances of development of multiple antimicrobial resistance caused by uropathogens and leads to more side effects (such as liver and renal damage and *Clostridium difficile* infection) (33). This could increase the severity of UTIs in the elderly and make their treatment more difficult.

In this study, the number of UTI cases and its incidence rate among females were significantly higher than men of all age groups at a global level. These differences could be attributable to widely known anatomical differences between the sexes. Despite the differences, a common observation in the incidence rate among males and females is the increasing trend observed in the sexually active puberty age and child-bearing groups and in the advanced age group of 80 years. As previously reported (42), our data also show a slight decrease in UTIs in middle-aged (35–80 years) women. Interestingly, across all age groups, there was no sex-based difference in mortality rate and DALYs, which were very low until a significant increase occurred after the age of 65 to 75 years. These trends further prove that UTIs are not a deadly disease in younger patients. Our data underline that health policymakers should pay more attention and allocate more medical resources to the treatment of UTIs in elderly of both sexes.

This study has several limitations. First, the GBD estimation of UTIs is reconstructed through mathematical models based on a huge number of data sources of varying quality, which may (to some extent) deviate from the actual data, particularly in some underdeveloped areas where a priori information is extremely scarce, such as the Caribbean, South Asia, and Africa (13). Second, because of the high rate of missed diagnosis in developing countries, estimates of the burden are inevitably biased. Third, the study lacks relevant data on the anatomic sites, causative pathogens, and antibiotic treatment. Fourth, the global data on urinary instrumentation was not available, and its contribution to the trend of UTI burden needs to be further studied.

## Conclusion

Despite these limitations, this study presents a comprehensive picture of the global burden of UTIs according to socio-demographic status, region, sex, and age, over the last three decades based on the GBD 2019 study. Although the global burden rate in UTIs-related incidence and DALYs remained relatively stable, the corresponding mortality rate increased by 0.55% annually, which led to the global deaths due to UTIs in 2019 was 2.4-fold of that in 1990. The disease burden among elderly people typically increased with age, and the annual increasing trend was more obvious in people over 60 years of age, especially in higher SDI regions. Given the population is aging and growing, we believe the global burden of UTIs will be heavier without appropriate interventions, which underlines a growing public health challenge for UTIs. Our research results could be useful for policymakers in terms of effectively allocating cost-effective, preventive, and treatment solutions to mitigate burdens, such as appropriate patient education, behavior building, perineal care, and antimicrobial agent usage, especially in higher SDI regions and elderly population.

## Data availability statement

Publicly available datasets were analyzed in this study. This data can be found at: http://ghdx.healthdata.org/gbd-results-tool.

## Ethics statement

The studies involving human participants were reviewed and approved by Institutional Review Boards of Qilu Hospital of Shandong University with approval number KYLL-202011(KS)-239. Written informed consent for participation was not required for this study in accordance with the national legislation and the institutional requirements.

## Author contributions

Conceptualization: FY and HW (equal). Formal analysis and methodology: XY, HC, and HW (equal). Data curation: XY, HC, YZ, and SQ (equal). Funding: FY, HW, and XY (equal). Visualization: XY and HC (lead). Supervision: FY (lead). Validation: HC, YZ, and SQ (equal). Writing—original draft: XY and HW (lead). Writing—review and editing: All authors (equal). All authors gave final approval and agreed to be accountable for all aspects of the work ensuring integrity and accuracy.

## Funding

This work was supported by grants from the National Natural Science Foundation of China (81873927, 82072231, 91949202, 82090020, 82090024, and 82103912), Taishan Scholars Program of Shandong Province (tsqn202103165), Clinical Research Center of Shandong University (2020SDUCRCC013), Shandong Provincial Natural Science Foundation (ZR2020QH302), and China Postdoctoral Science Foundation (2018M632685 and 2021M700080). The funders were not involved in the collection, analysis, or interpretation of data, or the writing or submission of this report.

## Conflict of interest

The authors declare that the research was conducted in the absence of any commercial or financial relationships that could be construed as a potential conflict of interest.

## Publisher's note

All claims expressed in this article are solely those of the authors and do not necessarily represent those of their affiliated organizations, or those of the publisher, the editors and the reviewers. Any product that may be evaluated in this article, or claim that may be made by its manufacturer, is not guaranteed or endorsed by the publisher.
