# Supplementary material for: Disease burden and long-term trends of urinary tract infections: A worldwide report
Source: Front Public Health. 2022 Jul 27;10:888205. doi: 10.3389/fpubh.2022.888205 (PMC9363895; doi:10.3389/fpubh.2022.888205)
Supplement: Supplementary file 1 [file Data_Sheet_1.PDF]

Supplementary Materials

Disease Burden and Long-term Trends of Urinary Tract Infections: A World-wide Report

The supplementary material includes detailed information of methods, report checklist, 3 supplementary tables and 5 supplementary figures.

- Detailed Information of Methods
- GATHER checklist of information that should be included in reports of global health estimates.
- Table S1: Incidence and age-standardized incidence rate per 1000 people for urinary tract infections in 1990 and 2019, and its estimated annual percentage change from 1990 to 2019.
- Table S2: DALYs and age-standardized DALY rate per 1000 people for urinary tract infections in 1990 and 2019, and its estimated annual percentage change from 1990 to 2019.
- Table S3. Age-standardized burden rate in 2019 for urinary tract infections in 2019, and its estimated annual percentage change from 1990 to 2019 in 204 countries and territories.
- Figure S1. Global incidence and DALYs of urinary tract infection for both sexes across 204 countries and territories.
- Figure S2. Change in the incidence of urinary tract infection across all age groups and in both sexes from 1990 to 2019.
- Figure S3. Changes in DALYs associated with urinary tract infection across all age groups and in both sexes from 1990 to 2019.
- Figure S4. Factors associated the EAPC in ASDR associated with urinary tract infection in both sexes from 1990 to 2019.
- Figure S5. Factors associated with EAPC in the ASIR of urinary tract infection in both sexes from 1990 to 2019.

Detailed Information of Methods

Overview

The Global Burden of Diseases, Injuries, and Risk Factors Study (GBD) 2019 is a multinational collaborative research study that estimates 369 diseases and injuries burden in 204 countries and territories in the world[1]. The study is an ongoing effort, updated annually, and is designed to allow for consistent comparison over time from 1990 to 2019, by age and sex, and across locations. The study produces standard epidemiological measures such as incidence, prevalence, and mortality information as well as summary measures of health, including years of life lost (YLLs), years lived with disability (YLDs), and disability-adjusted life-years (DALYs). DALYs represent the sum of years of life lost prematurely and years lived with disability; can be estimated from life tables, estimates of prevalence, and disability weights; and may be expressed as counts or rates. Annual updates to the study include new diseases, new data sources, and updates to methods. Input data in the GBD 2019 study were extracted from censuses, household surveys, civil registration and vital statistics, disease registries, health service use, air pollution monitors, satellite imaging, disease notifications, and other sources. Each of these types of data are identified from systematic review of published studies, searches of government and international organization websites, published reports, primary data sources such as the Demographic and Health Surveys, and contributions of datasets by GBD collaborators. Cause-specific death rates and cause fractions were calculated using the Cause of Death Ensemble model and spatiotemporal Gaussian process regression. Cause-specific deaths were adjusted to match the total all-cause deaths calculated as part of the GBD population, fertility, and mortality estimates. A Bayesian meta-regression modelling tool, DisMod-MR 2.1, was used to ensure consistency between incidence, prevalence, remission, excess mortality, and cause-specific mortality for most causes. All results are available via the GBD Compare website (https://vizhub.healthdata.org/gbd-compare/), and all input data is identified via the Global Health Data Exchange website (http://ghdx.healthdata.org/). The detailed

original data introduction, analysis methods, repeated codes of the GBD 2019 study have been reported in the supplemental appendix of previous articles[1,2], supporting website (http://ghdx.healthdata.org/gbd-2019) and summarized here.

Definition and Data source

Each step used to analyze the GBD database in the current study complied with the Guidelines for Accurate and Transparent Health Estimates Reporting (GATHER) statement[3], as supplementary report checklist. In the GBD methodology, urinary tract infection and interstitial nephritis (UTIs) was defined as a kidney infection that can lead to systemic symptoms such as fever and weakness and can cause discomfort and difficulty with daily activities. The GBD 2019 study attributes each death to a single underlying cause that began the series of events leading to death, in accordance with International Classification of Diseases and Injuries (ICD-9 and ICD-10), with 590-590.9, 595-595.9, 597-597.9, 599.0 in the ICD-9 and N10-N12.9, N13.6, N15, N15.1-N16.8, N30-N30.3, N30.8-N30.9, N34-N34.3, N39.0-N39.2 in the ICD-10 were considered as UTIs in the GBD 2019 study[1]. The standard CODEm approach with location-level covariates based on combined vital registration data were used to model the deaths due to UTIs. To estimate non-fatal health outcome of UTIs, GBD collected hospital discharges and claims data on UTIs across the world. According to the GBD inclusion criteria, a total of 311 original data sources related to UTIs were identified. The following process of adjusting for non-reference data using MR-BRT with the logit-transformation method was used for data processing: (1) Identify data points with overlapping year, age, sex, and location between claims (alternative case definition) and hospital discharges (reference case definition); (2) Logit transform overlapping data points of alternative and reference case definitions; (3) Convert overlapping data points into a difference in logit space using the following equation: $\text{logit(alterative)} - \text{logit(reference)}$; (4) Use the delta method to compute standard errors of overlapping data points in logit space, then calculate standard error of logit difference using the following equation:
$\sqrt{(\text{variance of alterative}) + (\text{variance of reference})}$; (5) Using MR-BRT, conduct a

random effects meta-regression to obtain the pooled logit difference of alternative to reference; (6) Apply the pooled logit difference to all data points of alternative case definitions using the following equation: $new_{estimate}$=inverse.logit(logit(alternative))-(pooled logit difference); (7) Calculate new standard errors using the delta method, accounting for gamma (between-study heterogeneity). The basis of the GBD disability weight survey assessments was lay descriptions of sequelae highlighting major functional consequences and symptoms. In GBD 2019 study, the GBD Collaborators estimated UTIs burden using a DisMod-MR Bayesian meta-regression model to produce estimates by age, sex, year, and country. The analysis process and reproducible statistical codes of the estimated UTIs can be collected from the following website: http://ghdx.healthdata.org/gbd-2019/code. We collected data on the burden of UTIs by gender and 5-year age group in 204 countries and territories from 1990 to 2019 from the online data repository of Institute for Health Metrics and Evaluation (http://ghdx.healthdata.org/gbd-resultstool).

In order to describe the disease burden of UTIs in different geographic units, the world was further divided geographically into 21 GBD regions such as high-income Asia-Pacific, Central Latin America, and Central Europe, which were also simplified into 7 super GBD regions such as high-income regions. In addition, 204 countries and territories are divided into five regions based on their Socio-demographic Index (SDI), namely, Low, Low-Middle, Middle, High-Middle and High SDI regions. Developed by GBD researchers and used to help produce these estimates, the SDI is a composite indicator of development status strongly correlated with health outcomes, as described elsewhere[1,4]. It is the geometric mean of 0 to 1 indices of total fertility rate under the age of 25, mean education for those ages 15 and older, and lag distributed income per capita. As a composite, a location with an SDI of 0 would have a theoretical minimum level of development relevant to health, while a location with an SDI of 1 would have a theoretical maximum level.

Flowchart

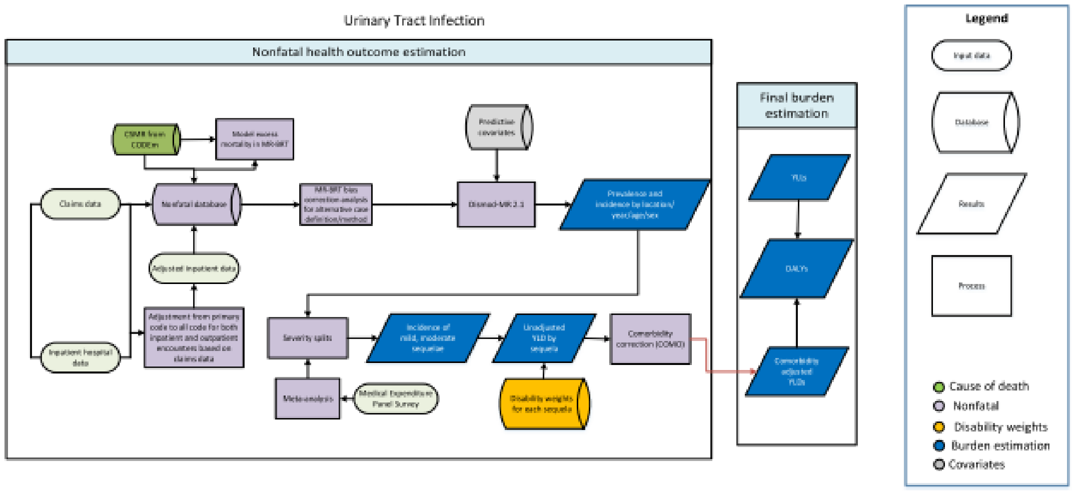

The flowchart of the input data and methodological summary for urinary tract infection

Statistical analysis

The age-standardized incidence rate (ASIR), age-standardized mortality rate (ASMR), and age-standardized DALYs rate (ASDR) were used to assess the differences in the burden of UTIs by historical periods, genders, and locations, to avoid differences caused by the age composition of the population. The age-standardized rates (ASRs) were calculated by the direct method, which sums up the products of the age-specific and the number of persons in the same age subgroup of the standard population then divides them by the sum of the standard population weights, based on the world population standard age structure by WHO 2001[5]. The 95% uncertainty intervals (UIs) of every metric in the GBD study were estimated based on the 25th and 975th ordered values of random 1000 draws of the corresponding posterior distribution[1]. We further computed the estimated annual percentage change (EAPC) to depict the secular trend in various ASRs of UTIs burden based on a regression model by fitting the natural logarithm of the ASR with the calendar year, namely, ln (ASR) = $\alpha + \beta*$ calendar year + ε[6-8]. The EAPC and its 95% confidence interval (CI) were estimated based on the formula of $100 \times (\exp(\beta) - 1)$. The age-standardized indicator was recognized to be in in an increasing trend when the EAPCs and the lower boundary of the 95% CI are positive; conversely, to be a decreasing trend when

EAPCs and the upper boundary of the 95% CI are negative. The ASRs of UTIs in 1990 could reflect the baseline disease reservoir, and the SDI in 2019 could denote the availability and level of health care of every country[7]. We explored the relationship between the EAPC and ASRs of UTIs in 1990 to test whether the high-burden countries pay more attention to control the UTIs burden, because the countries with low UTIs burden are unlikely to make the prevention and treatment of UTIs as a high priority due to limited economic conditions, especially in poorer regions. Moreover, we also could identify the key countries with high baseline burden and overall increasing trend. We applied the Spearman rank correlation to estimate the relationship of the EAPCs in UTIs burden with the baseline burden in 1990 and the SDI in 2019 in 204 countries and territories, because of the non-normal distribution of data[8]. Taking into account the possible non-linear relationship, the local weighted scatter plot smoothing (LOWESS) regression was used to display more detailed information between the EAPC of ASR and possible factors. p, which was implemented using the geom_smooth function with default parameters of ggplot2 package. All statistical analyses in this study were conducted using R program version 4.0.3 (https://www.Rproject.org/), and the two-sided P value <0.05 was considered statistically significant.

Reference

[1] GBD 2019 Diseases and Injuries Collaborators. Global burden of 369 diseases and injuries in 204 countries and territories, 1990-2019: a systematic analysis for the Global Burden of Disease Study 2019. Lancet 2020, 396(10258): 1204-1222. https://doi.org/10.1016/s0140-6736(20)30925-9

[2] GBD 2019 Risk Factors Collaborators. Global burden of 87 risk factors in 204 countries and territories, 1990-2019: a systematic analysis for the Global Burden of Disease Study 2019. Lancet 2020, 396(10258): 1223-1249. https://doi.org/10.1016/s0140-6736(20)30752-2

[3] Stevens GA, Alkema L, Black RE, Boerma JT, Collins GS, Ezzati M, et al. Guidelines for Accurate and Transparent Health Estimates Reporting: the GATHER statement. Lancet 2016, 388(10062): e19-e23. https://doi.org/10.1016/s0140-6736(16)30388-9

[4] GBD 2017 Disease and Injury Incidence and Prevalence Collaborators. Global, regional, and national incidence, prevalence, and years lived with disability for 354 diseases and injuries for 195 countries and territories, 1990-2017: a systematic analysis for the Global Burden of Disease Study 2017. Lancet 2018, 392(10159): 1789-1858. https://doi.org/10.1016/s0140-6736(18)32279-7

[5] Lozano R, Naghavi M, Foreman K, Lim S, Shibuya K, Aboyans V, et al. Global and regional mortality from 235 causes of death for 20 age groups in 1990 and 2010: a systematic analysis for the Global Burden of Disease Study 2010. Lancet 2012, 380(9859): 2095-2128. https://doi.org/10.1016/s0140-6736(12)61728-0

[6] Hankey BF, Ries LA, Kosary CL, Feuer EJ, Merrill RM, Clegg LX, et al. Partitioning linear trends in age-adjusted rates. Cancer Causes Control 2000, 11(1): 31-35. https://doi.org/10.1023/a:1008953201688

[7] Liu Z, Jiang Y, Yuan H, Fang Q, Cai N, Suo C, et al. The trends in incidence of primary liver cancer caused by specific etiologies: Results from the Global Burden of Disease Study 2016 and implications for liver cancer prevention. J Hepatol 2019, 70(4): 674-683. https://doi.org/10.1016/j.jhep.2018.12.001

[8] Yang X, Fang Y, Chen H, Zhang T, Yin X, Man J, et al. Global, regional and national burden of anxiety disorders from 1990 to 2019: results from the Global Burden of Disease Study 2019. Epidemiol Psychiatr Sci 2021, 30: e36. https://doi.org/10.1017/s2045796021000275

GATHER checklist of information that should be included in reports of global health estimates

#	Checklist item	Section/paragraph/ interpretation
**Objectives and funding**		
1	Define the indicators, populations, and time periods for which estimates were made.	Methods / “Data Sources”
2	List the funding sources for the work.	Funding
**Data Inputs**		
For all data inputs from multiple sources that are synthesized as part of the study:		
3	Describe how the data were identified and how the data were accessed.	As mentioned in the Methods / “Data Sources” section, the details have been published previously.
4	Specify the inclusion and exclusion criteria. Identify all ad-hoc exclusions.	As mentioned in the Methods / “Data Sources” section, the details have been published previously.
5	Provide information on all included data sources and their main characteristics. For each data source used, report reference information or contact name/institution, population represented, data collection method, year(s) of data collection, sex and age range, diagnostic criteria or measurement method, and sample size, as relevant.	Available via online data source tools (http://ghdx.healthdata.org/gbd-2019/data-input-sources).
6	Identify and describe any categories of input data that have potentially important biases (e.g., based on characteristics listed in item 5).	As mentioned in the Methods / “Data Sources” section, the details have been published previously.
For data inputs that contribute to the analysis but were not synthesized as part of the study:		
7	Describe and give sources for any other data inputs.	Available via online data source tools (http://ghdx.healthdata.org/gbd-2019/data-input-sources).
For all data inputs:		
8	Provide all data inputs in a file format from which data can be efficiently extracted (e.g., a spreadsheet as opposed to a PDF), including all relevant meta-data listed in item 5. For any data inputs that cannot be shared due to ethical or legal reasons, such as third-party ownership, provide a contact name or the name of the institution that retains the right to the data.	Available via online data source tools (http://ghdx.healthdata.org/gbd-2019/data-input-sources); input data not available in tools will be made available upon request.
**Data analysis**		
9	Provide a conceptual overview of the data analysis method. A diagram may be helpful.	Flow diagrams of the overall methodological processes were available online (http://ghdx.healthdata.org/gbd-2019/code/)

10	Provide a detailed description of all steps of the analysis, including mathematical formulae. This description should cover, as relevant, data cleaning, data pre-processing, data adjustments and weighting of data sources, and mathematical or statistical model(s).	As mentioned in the Methods / “Statistical Analysis” section, the details have been published previously.
11	Describe how candidate models were evaluated and how the final model(s) were selected.	As mentioned in the Methods / “Statistical Analysis” section, the details have been published previously.
12	Provide the results of an evaluation of model performance, if done, as well as the results of any relevant sensitivity analysis.	As mentioned in the Methods / “Statistical Analysis” section, the details have been published previously.
13	Describe methods for calculating uncertainty of the estimates. State which sources of uncertainty were, and were not, accounted for in the uncertainty analysis.	Methods / “Statistical Analysis” section
14	State how analytic or statistical source code used to generate estimates can be accessed.	Methods / “Statistical Analysis” section
**Results and Discussion**		
15	Provide published estimates in a file format from which data can be efficiently extracted.	Results, and online data tools (data visualization tools, and data query tools, http://ghdx.healthdata.org/gbd-2019)
16	Report a quantitative measure of the uncertainty of the estimates (e.g. uncertainty intervals).	Results, and online data tools (data visualization tools, and data query tools, http://ghdx.healthdata.org/gbd-2019)
17	Interpret results in light of existing evidence. If updating a previous set of estimates, describe the reasons for changes in estimates.	Discussion, paragraphs 2-5
18	Discuss limitations of the estimates. Include a discussion of any modelling assumptions or data limitations that affect interpretation of the estimates.	Discussion, paragraph 6

Table S1: Incidence and age-standardized incidence rate per 1000 people for urinary tract infections in 1990 and 2019, and its estimated annual percentage change from 1990 to 2019.

Characteristics	1990		2019		EAPC of ASIR (95%CI)
	ASIR/1000 (95%UI)	Incident cases*10^6 (95%UI)	ASIR/1000 (95%UI)	Incident cases*10^6 (95%UI)	from 1990 to 2019
**Global**	49.9 (44.34, 54.99)	252.25 (223.31, 279.3)	50.76 (45.17, 55.94)	404.61 (359.43, 446.55)	0.08 (0.04, 0.12)
Male	19.85 (17.84, 21.75)	48.85 (43.72, 53.69)	22.12 (19.89, 24.2)	87.19 (78.01, 95.42)	0.39 (0.37, 0.41)
Female	79.9 (70.65, 88.44)	203.4 (179.55, 225.94)	79.46 (70.45, 87.92)	317.42 (280.97, 351.26)	0 (-0.05, 0.04)
**SDI region**					
High SDI	68.68 (61.11, 75.87)	59.93 (53.12, 66.34)	64.24 (57.9, 70.27)	72.95 (65.89, 79.7)	-0.25 (-0.31, -0.18)
High-middle SDI	49.65 (44.45, 54.7)	57.74 (51.51, 63.57)	46.28 (41.35, 50.89)	71.53 (63.44, 78.82)	-0.28 (-0.33, -0.24)
Middle SDI	37.31 (32.95, 41.3)	60.11 (53.11, 66.79)	43.92 (38.98, 48.55)	110.65 (97.37, 122.39)	0.63 (0.6, 0.66)
Low-middle SDI	55.57 (48.73, 61.99)	54.49 (47.54, 60.93)	59.23 (52.21, 65.69)	102.77 (90.03, 114.43)	0.27 (0.25, 0.28)
Low SDI	46.7 (40.79, 52.09)	19.81 (17.22, 22.23)	46.79 (40.86, 51.97)	44.06 (38.2, 49.53)	0.12 (0.08, 0.15)
**GBD Region**					
High-income Asia Pacific	61.71 (55.78, 67.8)	10.62 (9.56, 11.7)	63.42 (57.52, 69.15)	11.97 (10.92, 13.06)	-0.04 (-0.11, 0.03)
High-income North America	71.72 (64.19, 78.78)	22.01 (19.61, 24.21)	67.69 (62.34, 72.98)	30.12 (27.76, 32.74)	-0.3 (-0.36, -0.24)
Western Europe	78.19 (67.77, 88.2)	32 (27.81, 36.08)	71.96 (61.92, 81.37)	31.83 (27.69, 35.61)	-0.28 (-0.41, -0.14)
Australasia	95.27 (82.91, 107.53)	2.02 (1.75, 2.29)	96.63 (83.65, 108.69)	2.96 (2.58, 3.31)	0.09 (0.06, 0.11)
Southern Latin America	72.31 (63.1, 81.5)	3.52 (3.07, 3.98)	74.07 (64.15, 82.5)	5.25 (4.57, 5.85)	0.12 (0.04, 0.19)
Andean Latin America	122.16 (107.45, 136.61)	4.18 (3.67, 4.68)	131.64 (115.55, 147.41)	8.41 (7.36, 9.46)	0.45 (0.4, 0.51)
Tropical Latin America	130.5 (114.64, 145.87)	18.69 (16.33, 20.98)	130.86 (114.9, 146.09)	30.97 (27.13, 34.61)	0.03 (-0.05, 0.11)
Central Latin America	68.1 (61, 75.29)	10.5 (9.4, 11.67)	78.78 (71.21, 86.95)	19.5 (17.56, 21.52)	0.48 (0.29, 0.67)
Caribbean	86.48 (76.48, 96.96)	2.95 (2.59, 3.35)	84.17 (74.08, 93.76)	4.07 (3.59, 4.55)	-0.08 (-0.08, -0.07)
Eastern Europe	85.71 (76.6, 94.77)	19.78 (17.57, 21.84)	85.94 (76.57, 95.08)	18.84 (16.66, 20.97)	0.03 (0.02, 0.04)

Central Europe	48.51 (44.07, 52.96)	6.03 (5.48, 6.61)	44.49 (40.63, 48.5)	5.72 (5.17, 6.3)	-0.28 (-0.37, -0.2)
Central Asia	59.46 (53.63, 65.28)	3.94 (3.54, 4.34)	59.71 (53.5, 65.9)	5.6 (4.98, 6.2)	0.03 (0.01, 0.04)
North Africa and Middle East	43.07 (38.12, 47.96)	14.62 (12.87, 16.46)	43.86 (38.56, 48.87)	27.48 (23.89, 30.73)	0.09 (0.07, 0.1)
South Asia	64.77 (56.63, 72.13)	61.64 (53.55, 69.13)	70.54 (61.96, 78.36)	126.65 (110.51, 141.44)	0.33 (0.3, 0.35)
Southeast Asia	24.55 (21.32, 27.45)	9.57 (8.26, 10.69)	23.63 (20.55, 26.43)	16.81 (14.56, 18.9)	-0.2 (-0.27, -0.13)
East Asia	12.77 (11, 14.38)	14.97 (12.84, 16.83)	12.31 (10.73, 13.81)	23.19 (20.19, 26.28)	-0.19 (-0.27, -0.12)
Oceania	18.22 (15.8, 20.46)	0.09 (0.08, 0.1)	18.29 (15.71, 20.8)	0.21 (0.18, 0.24)	-0.02 (-0.04, -0.01)
Western Sub-Saharan Africa	38.13 (33.36, 42.72)	6.05 (5.27, 6.82)	39.92 (34.99, 44.52)	15.26 (13.21, 17.17)	0.16 (0.15, 0.17)
Eastern Sub-Saharan Africa	34.35 (30.13, 38.44)	5.04 (4.37, 5.68)	35.21 (30.67, 39.2)	11.84 (10.23, 13.39)	0.09 (0.08, 0.11)
Central Sub-Saharan Africa	33.47 (28.71, 37.72)	1.54 (1.33, 1.76)	34.3 (29.64, 38.87)	3.89 (3.35, 4.43)	0.08 (0.07, 0.09)
Southern Sub-Saharan Africa	50.77 (43.41, 57.16)	2.45 (2.1, 2.77)	50.85 (44.07, 56.99)	4.04 (3.48, 4.55)	0.01 (-0.01, 0.03)

ASIR, age-standardized incidence rate; No., number; UI, uncertainty interval; EAPC, estimated annual percentage change; CI, confidential interval.

Table S2: DALYs and age-standardized DALY rate per 1000 people for urinary tract infections in 1990 and 2019, and its estimated annual percentage change from 1990 to 2019.

Characteristics	1990		2019		EAPC (95%CI)
	ASDR/100 000 (95%UI)	DALYs*10^5 (95%UI)	ASDR/100 000 (95%UI)	DALYs*10^5 (95%UI)	from 1990 to 2019
**Global**	67.73 (59.96, 73.45)	30.8 (26.52, 33.82)	66.17 (56.56, 72.5)	52.02 (44.54, 57.05)	-0.08 (-0.11, -0.04)
Male	69.74 (59.16, 78.72)	14.72 (12.25, 16.56)	65.64 (52.8, 73.33)	24.08 (19.49, 27.06)	-0.17 (-0.21, -0.12)
Female	67.42 (58.34, 74.51)	16.08 (13.62, 18.07)	67.21 (57.89, 74.67)	27.94 (24.03, 30.99)	-0.04 (-0.09, 0.01)
**SDI region**					
High SDI	39.96 (36.42, 45.93)	4.03 (3.68, 4.66)	42.21 (36.29, 45.8)	8.08 (6.87, 8.75)	0.34 (0.19, 0.48)
High-middle SDI	50.28 (44.2, 53.45)	5.42 (4.73, 5.77)	48.05 (42.17, 52.04)	8.95 (7.81, 9.7)	-0.16 (-0.27, -0.06)
Middle SDI	52.26 (46.03, 56.51)	6.87 (5.95, 7.45)	50.88 (44.77, 55.65)	12.1 (10.6, 13.19)	-0.09 (-0.12, -0.06)
Low-middle SDI	116.89 (91.56, 133.68)	10.49 (7.86, 12.4)	110.74 (85.51, 127.52)	16.52 (12.75, 18.97)	-0.22 (-0.28, -0.16)
Low SDI	100.32 (85.15, 118.66)	3.97 (3.21, 4.84)	86.86 (72.95, 103.86)	6.34 (5.33, 7.6)	-0.55 (-0.59, -0.5)
**GBD Region**					
High-income Asia Pacific	23.13 (20.09, 33)	0.41 (0.36, 0.59)	24.09 (19.67, 27.37)	1.11 (0.88, 1.25)	0.49 (0.28, 0.7)
High-income North America	53.22 (47.24, 57.22)	1.89 (1.67, 2.02)	54.87 (49.37, 59.69)	3.38 (3.04, 3.68)	0.02 (-0.19, 0.23)
Western Europe	29.98 (26.7, 38.86)	1.63 (1.47, 2.12)	39.75 (30.31, 43.98)	3.84 (2.82, 4.22)	1.38 (1.11, 1.66)
Australasia	32.44 (28.79, 36.82)	0.07 (0.06, 0.08)	33.28 (28.23, 37.79)	0.16 (0.14, 0.18)	0.17 (-0.07, 0.4)
Southern Latin America	39.87 (35.94, 53.2)	0.18 (0.16, 0.24)	115.15 (74.47, 127.34)	0.96 (0.61, 1.06)	4.12 (3.58, 4.66)
Andean Latin America	71.13 (63.19, 87.41)	0.22 (0.19, 0.26)	75.84 (54.77, 95)	0.43 (0.32, 0.54)	0.58 (0.16, 1)
Tropical Latin America	107.61 (99.04, 133.17)	1.24 (1.14, 1.48)	167.29 (114.4, 183.79)	3.83 (2.62, 4.2)	2.28 (1.98, 2.57)
Central Latin America	66.87 (61.88, 78.94)	0.76 (0.7, 0.9)	90.34 (73.45, 104.18)	2.14 (1.74, 2.46)	1.69 (1.36, 2.02)
Caribbean	41.52 (36.46, 51.4)	0.13 (0.11, 0.16)	67.55 (51.67, 80.22)	0.33 (0.26, 0.4)	1.91 (1.68, 2.14)
Eastern Europe	86.7 (66.07, 94.65)	2.31 (1.76, 2.52)	80.85 (71.16, 93.34)	2.46 (2.16, 2.84)	-0.86 (-1.16, -0.57)
Central Europe	67.08 (48.9, 72.5)	0.95 (0.68, 1.02)	33.59 (29.04, 41.66)	0.66 (0.57, 0.82)	-2.13 (-2.78, -1.48)

Central Asia	83.07 (74.28, 88.21)	0.49 (0.45, 0.53)	137.71 (115.62, 155.19)	1.2 (1.01, 1.36)	1.7 (1.31, 2.09)
North Africa and Middle East	24.25 (20.21, 34.14)	0.61 (0.53, 0.74)	21.3 (18.53, 25.89)	0.95 (0.83, 1.12)	-0.5 (-0.7, -0.29)
South Asia	146.62 (115.66, 168.52)	12.44 (9.36, 14.79)	130.35 (99.88, 150.84)	19.97 (15.28, 23.12)	-0.49 (-0.56, -0.42)
Southeast Asia	82.49 (59.86, 93.64)	2.8 (2.03, 3.21)	71.52 (52.77, 86.15)	4.45 (3.24, 5.25)	-0.64 (-0.73, -0.55)
East Asia	22.62 (18.06, 25.9)	2.25 (1.77, 2.57)	12.33 (10.64, 15.16)	2.24 (1.92, 2.77)	-2.35 (-2.63, -2.06)
Oceania	70.08 (56.1, 90.58)	0.03 (0.02, 0.04)	64.34 (51.17, 80.51)	0.06 (0.05, 0.08)	-0.3 (-0.33, -0.26)
Western Sub-Saharan Africa	69.22 (56.59, 87.53)	1.12 (0.86, 1.47)	53.9 (41.58, 76.62)	1.78 (1.37, 2.48)	-1.02 (-1.11, -0.93)
Eastern Sub-Saharan Africa	77.39 (51, 117.29)	0.91 (0.64, 1.37)	60.91 (35.39, 96.12)	1.42 (0.87, 2.22)	-0.91 (-0.95, -0.87)
Central Sub-Saharan Africa	67.41 (46.01, 101.67)	0.23 (0.16, 0.37)	58.18 (34.46, 94.69)	0.42 (0.26, 0.69)	-0.59 (-0.67, -0.52)
Southern Sub-Saharan Africa	34.53 (24.43, 40.03)	0.13 (0.1, 0.16)	31.57 (25.23, 38.66)	0.21 (0.16, 0.26)	-0.39 (-1.08, 0.31)

DALYs, disability-adjusted life years; ASDR, age-standardized DALYs rate; No., number; UI, uncertainty interval; EAPC, estimated annual percentage change; CI, confidential interval.

Table S3. Age-standardized burden rate in 2019 for urinary tract infections in 2019, and its estimated annual percentage change from 1990 to 2019 in 204 countries and territories.

Location name	ASIR/1000 in 2019 (95%UI)	EAPC (95%CI) in ASIR, 1990-2019	ASMR/100 000 in 2019 (95%UI)	EAPC (95%CI) in ASMR, 1990-2019	ASDR/100 000 in 2019 (95%UI)	EAPC (95%CI) in ASDR, 1990-2019
Paraguay	135.59 (114.43, 155.69)	-0.013 (-0.039, 0.014)	4.69 (2.64, 6.29)	3.828 (3.242, 4.416)	88.14 (56.52, 115.5)	2.945 (2.493, 3.399)
Ecuador	155.43 (137.08, 174.01)	1.094 (0.944, 1.245)	2.75 (1.65, 3.49)	3.024 (2.517, 3.535)	51.72 (35.34, 64.39)	1.987 (1.635, 2.34)
Brazil	130.75 (114.9, 145.78)	0.038 (-0.045, 0.121)	9.49 (5.79, 10.57)	3.483 (3.106, 3.861)	169.32 (115.84, 186.08)	2.258 (1.952, 2.565)
Peru	125.21 (106.24, 143.27)	0.157 (0.13, 0.185)	4.35 (2.87, 5.95)	1.429 (0.648, 2.217)	83.24 (57.8, 110.74)	0.396 (-0.26, 1.057)
Bolivia	114.84 (98.93, 134.11)	0.12 (0.085, 0.156)	5 (3.55, 6.41)	1.178 (1.082, 1.275)	91.69 (67.32, 114.96)	0.254 (0.176, 0.331)
Norway	109.1 (94.32, 124.45)	-0.146 (-0.233, -0.058)	3.7 (2.28, 4.19)	0.906 (0.601, 1.212)	46.87 (33.28, 53.24)	0.315 (0.075, 0.556)
Bermuda	98.64 (85.77, 111.76)	0.021 (0.013, 0.03)	1.52 (1.11, 1.9)	1.898 (1.486, 2.311)	29.31 (22.8, 35.82)	1.096 (0.845, 1.348)
New Zealand	107.64 (95.86, 119.96)	0.468 (0.389, 0.548)	2.06 (1.7, 2.4)	0.039 (-0.634, 0.718)	32.26 (27.99, 37.07)	-0.393 (-0.95, 0.166)
Puerto Rico	96.55 (84.7, 109.03)	0.048 (0.024, 0.072)	4.43 (2.84, 5.69)	2.425 (1.463, 3.396)	80.12 (57.91, 102.09)	2.123 (1.331, 2.921)
Australia	94.69 (81.54, 107.68)	0.012 (-0.009, 0.034)	2.3 (1.82, 2.62)	1.082 (0.565, 1.602)	33.47 (28.17, 38.09)	0.322 (-0.031, 0.676)
Antigua and Barbuda	90.96 (78.65, 102.84)	-0.027 (-0.049, -0.005)	4.81 (3.68, 5.69)	3.986 (3.514, 4.46)	87.14 (69.62, 102.92)	3.205 (2.817, 3.596)
Cuba	90.77 (79.26, 101.95)	-0.011 (-0.02, -0.002)	1.57 (1.15, 1.94)	3.903 (3.578, 4.229)	36.6 (26.47, 45.43)	2.811 (2.596, 3.026)
Virgin Islands US	90.94 (79.87, 102.97)	0.061 (0.041, 0.082)	5.71 (4.35, 6.76)	1.854 (1.585, 2.124)	100.99 (79.7, 119.98)	1.455 (1.203, 1.707)
Barbados	91.38 (79.27, 103.78)	0.067 (0.054, 0.081)	12.02 (8.5, 14.55)	3.721 (3.003, 4.444)	215.91 (155.17, 264.92)	3.184 (2.561, 3.81)
Finland	90 (75.62, 103.26)	-0.008 (-0.097, 0.081)	1.45 (1.16, 2.15)	-4.942 (-5.66, -4.218)	23.04 (18.95, 33.11)	-4.347 (-4.866, -3.825)
Jamaica	87.94 (75.23, 99.15)	-0.034 (-0.045, -0.022)	4.69 (3.03, 6.06)	3.041 (2.22, 3.869)	87.2 (60.64, 111.31)	2.701 (1.977, 3.431)
Estonia	86.7 (76.26, 96.62)	-0.121 (-0.137, -0.105)	1.91 (1.44, 2.69)	-4.424 (-4.852, -3.993)	44.42 (33.59, 65.96)	-4.506 (-4.896, -4.115)
Lithuania	86.94 (76.18, 98.09)	0.116 (-0.399, 0.633)	2.3 (1.83, 2.81)	-0.279 (-0.711, 0.154)	55.14 (44.97, 68.58)	-0.66 (-1.003, -0.316)
Saint Lucia	86.25 (75.23, 97.36)	-0.019 (-0.036, -0.002)	4.92 (3.56, 6.02)	2.826 (2.422, 3.233)	92.71 (66.58, 112.34)	2.661 (2.338, 2.985)
Latvia	85.36 (75.1, 95.5)	0.394 (0.018, 0.771)	2.71 (2.17, 3.34)	-1.894 (-2.443, -1.343)	63.53 (51.48, 80.08)	-2.213 (-2.688, -1.737)
Ukraine	85.76 (74.94, 96.42)	-0.088 (-0.117, -0.059)	2.23 (1.81, 3.13)	-0.357 (-0.691, -0.022)	75.08 (62.15, 93.78)	-0.548 (-0.951, -0.143)
Sweden	85.99 (75.15, 98.76)	-0.019 (-0.082, 0.044)	2.14 (1.79, 2.47)	-1.464 (-1.854, -1.073)	30.61 (26.69, 35.87)	-1.575 (-1.875, -1.274)

Belarus	85.84 (74.23, 96.59)	-0.025 (-0.041, -0.009)	2.69 (2.06, 3.82)	-0.522 (-1.08, 0.04)	70.65 (54.22, 97.83)	-1.109 (-1.625, -0.591)
Germany	86.27 (72.5, 100.04)	0.092 (0.064, 0.12)	2.49 (1.93, 2.83)	3.532 (3.202, 3.863)	39.21 (30.54, 44.62)	2.012 (1.754, 2.272)
Austria	91.26 (77.8, 105.73)	0.372 (0.273, 0.47)	1.37 (1.15, 1.84)	-0.582 (-1.5, 0.344)	24.8 (21.28, 30.77)	-1.068 (-1.754, -0.377)
Bahamas	86.28 (75.12, 97.76)	0.045 (0.026, 0.064)	6.3 (4.72, 7.72)	2.518 (2.091, 2.946)	130.74 (99.83, 162.15)	2.199 (1.843, 2.555)
Saint Kitts and Nevis	84.6 (73.46, 95.72)	-0.063 (-0.08, -0.045)	8.71 (5.96, 10.49)	2.836 (2.417, 3.258)	166.17 (117.29, 204.31)	2.255 (1.883, 2.629)
Grenada	83.2 (72.37, 93.6)	-0.059 (-0.072, -0.046)	5.7 (4.42, 6.57)	4.105 (3.56, 4.653)	112.26 (86.88, 128.87)	3.684 (3.259, 4.11)
Russia	86.06 (76.55, 94.93)	0.064 (0.049, 0.079)	3.57 (2.93, 4.14)	-0.439 (-0.892, 0.017)	84.92 (72.65, 99.09)	-0.918 (-1.247, -0.587)
Dominica	81.9 (71.77, 92.86)	-0.057 (-0.078, -0.036)	2.19 (1.46, 2.8)	3.621 (3.22, 4.024)	45.47 (32.15, 57.43)	2.65 (2.373, 2.929)
Trinidad and Tobago	84.37 (73.42, 95.63)	0.039 (0.024, 0.053)	3.55 (2.21, 4.77)	1.954 (1.491, 2.42)	75.37 (49.83, 102.34)	1.641 (1.241, 2.042)
Moldova	83.44 (72.56, 94.44)	-0.04 (-0.046, -0.033)	3.03 (2.56, 3.89)	-1.016 (-1.332, -0.698)	82.69 (70.58, 103.73)	-1.272 (-1.566, -0.978)
Saint Vincent and the Grenadines	81.93 (71.1, 93.21)	-0.027 (-0.052, -0.003)	6.68 (5.42, 7.82)	3.048 (2.84, 3.256)	130.75 (106.71, 153.6)	2.607 (2.381, 2.833)
Dominican Republic	82.63 (72.09, 92.49)	-0.056 (-0.067, -0.044)	0.95 (0.67, 1.26)	1.673 (1.427, 1.919)	24.6 (18.28, 31.81)	0.926 (0.765, 1.087)
Belize	84.14 (72.74, 95.66)	0.138 (0.119, 0.157)	5.53 (4.29, 6.51)	3.779 (2.894, 4.671)	115.75 (91.54, 135)	3.508 (2.735, 4.286)
Suriname	82.91 (71.5, 93.25)	0.076 (0.05, 0.102)	7.75 (4.79, 9.6)	3.084 (2.581, 3.59)	162.72 (104.83, 201.55)	2.682 (2.227, 3.14)
Denmark	81.99 (69.65, 94.6)	0.081 (0.053, 0.108)	3.44 (2.5, 3.97)	2.162 (1.426, 2.904)	44.07 (35.7, 49.66)	0.808 (0.271, 1.347)
Italy	52.37 (46.47, 58.1)	-2.609 (-3.115, -2.101)	0.95 (0.8, 1.12)	1.576 (1.007, 2.149)	15.63 (13.52, 17.95)	0.035 (-0.401, 0.474)
Guyana	79.64 (68.7, 91.96)	0.062 (0.04, 0.084)	5.2 (3.91, 6.54)	3.137 (2.608, 3.669)	113.48 (84.84, 145.71)	3.104 (2.624, 3.586)
Andorra	80.72 (68.42, 92.9)	0.162 (0.122, 0.202)	3.3 (2.45, 4.92)	-0.28 (-0.456, -0.103)	49.45 (37.69, 68.2)	-0.499 (-0.65, -0.348)
San Marino	80.24 (69.11, 92.55)	0.176 (0.155, 0.197)	1.21 (0.8, 1.88)	2.258 (1.905, 2.611)	23.41 (16.98, 31.26)	1.887 (1.588, 2.186)
Israel	76.4 (64.3, 89.19)	-0.003 (-0.014, 0.008)	5.52 (2.79, 6.52)	2.811 (1.547, 4.09)	68.67 (39.71, 79.27)	2.113 (1.081, 3.156)
Spain	76.53 (64.17, 89.36)	0.009 (-0.022, 0.039)	4.16 (2.5, 4.86)	2.95 (2.533, 3.369)	52.77 (35.09, 60.22)	1.985 (1.622, 2.35)
Greece	76.21 (64.12, 88.48)	0.033 (-0.001, 0.066)	1.64 (1.16, 1.88)	-0.315 (-1.832, 1.226)	23.45 (19, 26.67)	-0.455 (-1.559, 0.662)
Chile	76.26 (65.38, 86.84)	0.086 (-0.184, 0.356)	6.95 (5.34, 7.79)	0.619 (0.285, 0.953)	100.34 (80.86, 110.55)	0.14 (-0.152, 0.433)
Haiti	76.74 (64.93, 88.24)	0.073 (0.054, 0.092)	5.01 (3.11, 8.63)	1.254 (1.114, 1.394)	115.3 (75.76, 170.98)	0.799 (0.661, 0.938)
Iceland	75.51 (64.44, 87.86)	-0.26 (-0.346, -0.173)	1.5 (1.2, 1.79)	1.124 (0.658, 1.592)	23.41 (19.63, 27.57)	0.667 (0.331, 1.004)

Belgium	75.46 (63.92, 86.49)	-0.032 (-0.074, 0.01)	3.8 (1.7, 4.48)	4.897 (4.241, 5.558)	50.11 (26.12, 57.88)	3.769 (3.252, 4.288)
Netherlands	75.92 (64.09, 89.29)	0.061 (0.044, 0.077)	5.26 (3.13, 6.18)	0.123 (-0.478, 0.728)	61.29 (43.38, 69.54)	-0.224 (-0.805, 0.359)
Ireland	75.3 (64.79, 87.72)	0.072 (0.04, 0.104)	3.06 (2.54, 3.67)	-0.361 (-0.584, -0.138)	41.33 (35.87, 49.16)	-0.9 (-1.066, -0.734)
France	74.61 (63.22, 86.23)	0.043 (0.025, 0.061)	1.73 (1.37, 2.01)	-0.27 (-0.53, -0.01)	25.99 (22.19, 29.77)	-0.333 (-0.556, -0.11)
Luxembourg	73.66 (62.09, 85.23)	-0.024 (-0.183, 0.136)	0.98 (0.78, 1.19)	1.508 (1.229, 1.788)	17.24 (14.01, 20.81)	0.795 (0.633, 0.958)
Costa Rica	74.56 (66.1, 83.23)	0.084 (0.059, 0.109)	3.23 (2, 4.18)	4.063 (3.478, 4.651)	59.75 (41.29, 76.7)	3.27 (2.889, 3.652)
Uruguay	73.71 (64.2, 83.82)	0.107 (0.086, 0.128)	8.33 (4.4, 9.59)	5.717 (4.951, 6.489)	123.19 (70.89, 138.88)	4.801 (4.136, 5.471)
Monaco	73.1 (62.93, 84.44)	0.045 (0.029, 0.061)	0.52 (0.39, 0.78)	1.145 (0.871, 1.421)	12.27 (9.65, 15.7)	-0.427 (-0.563, -0.29)
USA	67.4 (62.25, 72.67)	-0.337 (-0.402, -0.272)	3.54 (3.08, 4)	-0.162 (-0.45, 0.126)	56.94 (51.34, 62.07)	-0.025 (-0.24, 0.19)
Argentina	73.23 (62.25, 82.33)	0.132 (0.113, 0.151)	7.97 (4.34, 9.2)	7.269 (6.258, 8.291)	120.77 (68.86, 136.06)	6.178 (5.378, 6.985)
Canada	70.56 (62.21, 78.31)	0.024 (0.007, 0.041)	2.65 (2.11, 3.05)	1.687 (1.288, 2.087)	37.83 (32.03, 42.28)	1.213 (0.915, 1.511)
Mexico	90.58 (81.84, 99.85)	0.874 (0.503, 1.245)	5.37 (4.39, 6.2)	1.865 (1.187, 2.548)	113.06 (91.91, 131.18)	1.878 (1.209, 2.551)
UK	54.42 (47.22, 61.47)	0.347 (-0.423, 1.124)	5.24 (3.6, 5.78)	3.955 (3.015, 4.904)	67.14 (48.47, 73.42)	3.054 (2.262, 3.851)
Bangladesh	71.5 (61.62, 81.14)	0.167 (0.138, 0.196)	2.48 (1.4, 3.22)	-0.208 (-0.464, 0.049)	67.53 (35.65, 86)	-1.142 (-1.303, -0.981)
Switzerland	68.56 (57.99, 78.57)	-0.117 (-0.504, 0.272)	2.11 (1.69, 2.44)	2.325 (1.626, 3.029)	29.42 (24.78, 33.53)	0.912 (0.561, 1.265)
Panama	68.81 (60.71, 77.46)	0.036 (0.02, 0.052)	2.52 (1.71, 3.25)	4.241 (3.732, 4.753)	52.57 (37.26, 67.82)	3.399 (3.008, 3.791)
Bhutan	70.9 (60.98, 80.33)	0.098 (0.08, 0.115)	5.49 (3.8, 7.22)	0.837 (0.766, 0.909)	118.65 (80.13, 157.99)	-0.155 (-0.222, -0.088)
Colombia	68.72 (61.1, 77.24)	0.103 (0.09, 0.115)	3.89 (2.81, 5.02)	2.437 (1.746, 3.133)	76.45 (58.13, 97.67)	1.772 (1.203, 2.343)
Portugal	63.16 (54.42, 72.71)	-0.333 (-0.413, -0.253)	5.36 (1.96, 6.33)	8.276 (7.315, 9.246)	69.88 (27.98, 81.15)	6.439 (5.639, 7.246)
Nicaragua	67.72 (60.36, 76.47)	0.058 (0.044, 0.072)	2.54 (1.89, 3.09)	1.372 (1.085, 1.66)	53.13 (37.77, 65.15)	0.459 (0.079, 0.84)
Venezuela	67.19 (59.97, 75.81)	0.013 (0.002, 0.024)	2.27 (1.58, 3.01)	2.691 (1.902, 3.486)	50.86 (36.79, 66.53)	2.136 (1.502, 2.774)
El Salvador	68.16 (59.22, 75.9)	0.198 (0.177, 0.219)	4.14 (3.03, 5.38)	-0.433 (-0.66, -0.206)	91.95 (68.08, 120.1)	-1.008 (-1.24, -0.776)
India	71.48 (62.9, 79.34)	0.372 (0.346, 0.398)	5.56 (4.31, 6.59)	-0.437 (-0.569, -0.306)	131.71 (102.84, 153.69)	-0.702 (-0.787, -0.616)
Armenia	66.84 (59.68, 74.85)	0.145 (0.128, 0.163)	9.73 (3.07, 13.1)	8.478 (7.228, 9.743)	190.42 (91.09, 248.45)	6.354 (5.327, 7.391)
Japan	67.15 (61.34, 73.01)	-0.055 (-0.16, 0.05)	1.54 (1.16, 1.74)	1.253 (0.868, 1.639)	24.41 (20.21, 27.83)	0.818 (0.568, 1.068)
Georgia	68.12 (62.29, 74.73)	0.25 (0.16, 0.341)	0.99 (0.69, 1.22)	4.07 (3.164, 4.984)	31.16 (24.3, 37.06)	2.246 (1.549, 2.948)

Honduras	64.02 (55.62, 72.46)	0.092 (0.084, 0.101)	4.04 (2.04, 5.89)	1.188 (0.949, 1.427)	77.31 (42.05, 115.08)	0.298 (0.099, 0.499)
Pakistan	65.02 (56.41, 72.51)	0.193 (0.176, 0.209)	6.77 (4.41, 8.57)	1.082 (0.766, 1.4)	173.86 (114.58, 218.49)	0.858 (0.588, 1.129)
Singapore	61.13 (54.64, 68.04)	0 (-0.023, 0.024)	5.79 (4.17, 6.71)	-1.489 (-2.031, -0.943)	82.07 (61.75, 92.73)	-1.984 (-2.512, -1.452)
Kyrgyzstan	60.57 (53.09, 67.15)	0.078 (0.052, 0.104)	4.06 (3.1, 4.71)	1.43 (0.325, 2.547)	129.13 (95.54, 150.75)	1.104 (0.055, 2.163)
Kazakhstan	60.01 (52.84, 67.7)	0.036 (0.019, 0.052)	3.02 (2.41, 4.31)	0.138 (-0.281, 0.558)	91.25 (72.42, 134.46)	-0.508 (-0.895, -0.119)
Guatemala	62.09 (54.37, 69.66)	0.183 (0.157, 0.209)	3.02 (2.18, 3.78)	3.178 (2.82, 3.537)	68.92 (48.13, 87.65)	2.696 (2.257, 3.136)
Azerbaijan	59.91 (52.34, 67.31)	0.019 (0.012, 0.026)	3.66 (2.64, 4.6)	3.59 (2.024, 5.18)	96.18 (73.1, 117.21)	2.065 (0.761, 3.387)
Uzbekistan	59.13 (52.09, 65.84)	0.035 (0.005, 0.064)	4.89 (4.06, 5.92)	2.536 (1.941, 3.135)	156.71 (127.83, 185.43)	1.806 (1.24, 2.374)
Tajikistan	57.85 (50.72, 64.7)	0.03 (0.007, 0.052)	8.48 (6.05, 10.48)	2.867 (2.44, 3.295)	242.35 (172.84, 301.91)	1.629 (1.13, 2.13)
Turkmenistan	57.87 (50.49, 65.44)	0.125 (0.084, 0.167)	5.71 (4.1, 7.22)	5.582 (4.593, 6.58)	205.59 (144.42, 260.33)	5.208 (4.287, 6.138)
Nepal	57.14 (49.69, 64.41)	-0.29 (-0.395, -0.184)	5.2 (3.45, 6.59)	1.342 (1.047, 1.638)	113.15 (76.34, 144.36)	0.313 (-0.013, 0.64)
Czech	55.28 (48.3, 61.59)	0.21 (0.133, 0.286)	2.59 (2.06, 3.15)	-2.315 (-3.1, -1.522)	45.14 (36.8, 55.58)	-2.925 (-3.62, -2.224)
South Korea	55.65 (49.03, 61.73)	0.011 (-0.004, 0.026)	1.43 (0.78, 2.09)	0.525 (-0.175, 1.23)	19.8 (12.68, 26.49)	-0.296 (-0.842, 0.253)
Mongolia	56.29 (49.08, 62.93)	0.082 (0.066, 0.098)	2.93 (2.15, 3.76)	-4.327 (-4.894, -3.757)	81.2 (62.49, 104.11)	-4.449 (-5.007, -3.888)
Malta	53.88 (45.98, 61.28)	-0.536 (-0.816, -0.256)	2.73 (2.16, 3.2)	1.196 (0.949, 1.444)	39.5 (31.85, 45.62)	0.825 (0.597, 1.052)
Greenland	55.67 (48.85, 62.7)	0.061 (0.041, 0.082)	7.16 (5.27, 8.68)	-0.706 (-1.043, -0.368)	104.13 (81.18, 124.67)	-1.017 (-1.328, -0.704)
Croatia	57.77 (51.88, 63.18)	0.606 (0.422, 0.789)	4.05 (2.53, 5)	2.244 (1.823, 2.666)	62.55 (47.29, 76.61)	1.199 (0.88, 1.519)
Cyprus	55.15 (47.02, 63.66)	-0.414 (-0.673, -0.155)	7.13 (2.83, 8.88)	0.084 (-0.378, 0.549)	76.51 (35.27, 93.07)	-0.475 (-0.864, -0.085)
Slovakia	52.82 (47.51, 58.67)	0.18 (-0.071, 0.432)	2.34 (1.62, 2.98)	-0.172 (-0.698, 0.357)	46.09 (34.51, 57.74)	-0.867 (-1.25, -0.483)
Slovenia	51.26 (46.23, 56.34)	-0.065 (-0.109, -0.022)	0.82 (0.5, 1.83)	-4.119 (-4.64, -3.595)	16.73 (11.6, 30.18)	-3.899 (-4.329, -3.468)
Botswana	52.25 (44.44, 59.54)	0.694 (0.333, 1.057)	1.83 (0.98, 2.77)	-0.042 (-0.362, 0.28)	45.2 (25.27, 67.19)	-0.012 (-0.394, 0.371)
Zimbabwe	51.09 (43.6, 57.98)	0.002 (-0.03, 0.035)	1.8 (0.99, 2.61)	1.198 (0.759, 1.638)	46.23 (26.01, 66.37)	1.406 (0.95, 1.864)
Brunei	51.51 (45.23, 57.62)	0.048 (0.006, 0.091)	10.62 (8.49, 13.02)	0.91 (0.643, 1.177)	148.27 (118.51, 174.31)	0.566 (0.245, 0.888)
Eswatini	51.27 (44.32, 58.94)	0.005 (-0.019, 0.029)	2.12 (1.1, 3.15)	-0.101 (-0.711, 0.514)	52.92 (28.91, 78.08)	0.123 (-0.522, 0.772)
South Africa	50.77 (43.92, 57.08)	-0.017 (-0.044, 0.01)	1.1 (0.93, 1.51)	-0.394 (-1.094, 0.31)	26.89 (22.87, 36.08)	-0.977 (-1.797, -0.151)
Namibia	52.14 (44.25, 59.62)	0.131 (0.1, 0.162)	1.55 (0.89, 2.23)	-0.911 (-1.332, -0.489)	36.09 (21.2, 51.22)	-0.897 (-1.338, -0.453)

Lesotho	50.13 (42.48, 57.95)	-0.022 (-0.05, 0.005)	2.55 (1.35, 3.74)	2.165 (1.833, 2.498)	63.15 (35.06, 89.78)	2.26 (1.905, 2.616)
Bosnia and Herzegovina	50.06 (45.08, 55.07)	-0.006 (-0.022, 0.011)	0.71 (0.48, 1.68)	-5.853 (-6.785, -4.912)	16.23 (11.62, 33.92)	-5.458 (-6.314, -4.594)
Hungary	48.94 (43.98, 53.98)	-0.013 (-0.035, 0.009)	1.76 (1.41, 2.39)	-2.366 (-2.848, -1.882)	34.93 (27.87, 49.45)	-2.612 (-3.018, -2.204)
Montenegro	48.53 (43.56, 53.36)	0 (-0.012, 0.011)	0.25 (0.2, 0.35)	0.096 (-0.016, 0.208)	8 (6.45, 10.15)	-0.319 (-0.431, -0.206)
Poland	35.88 (33.38, 38.66)	-1.311 (-1.516, -1.107)	1.86 (1.48, 2.2)	0.358 (-0.948, 1.681)	33.93 (27.78, 40.07)	-0.486 (-1.607, 0.648)
Kuwait	51.87 (45.21, 59.01)	0.273 (0.199, 0.347)	2.14 (1.17, 2.74)	5.793 (5.044, 6.547)	31.12 (19.25, 38.75)	4.045 (3.41, 4.684)
Palestine	47.87 (41.51, 54.18)	0.021 (-0.012, 0.054)	2.08 (1.36, 3.32)	-1.105 (-1.625, -0.583)	35.33 (22.89, 53.65)	-1.316 (-1.782, -0.849)
Lebanon	49.2 (42.42, 55.86)	0.159 (0.148, 0.169)	0.48 (0.24, 1.33)	0.127 (-0.07, 0.325)	11.42 (7.5, 22.92)	-0.111 (-0.283, 0.062)
Tunisia	48.44 (42.42, 54.56)	0.09 (0.081, 0.098)	0.48 (0.28, 1.33)	0.81 (0.638, 0.982)	11.35 (7.99, 22.46)	0.161 (0.023, 0.299)
Romania	47.27 (42.15, 52.4)	0.266 (0.184, 0.348)	1.43 (1.13, 2.23)	-1.816 (-2.149, -1.481)	33.18 (26.64, 49.18)	-2.37 (-2.758, -1.981)
Bulgaria	45.6 (40.64, 50.85)	-0.046 (-0.057, -0.035)	1.39 (0.91, 3.47)	-5.114 (-6.063, -4.155)	34.13 (23.63, 72.56)	-5.091 (-6.006, -4.168)
North Macedonia	46.42 (41.85, 51.76)	0.032 (0.021, 0.043)	0.19 (0.14, 0.37)	-2.382 (-3.119, -1.64)	6.82 (5.21, 9.47)	-1.991 (-2.474, -1.506)
Albania	46.73 (41.67, 52.18)	0.079 (0.071, 0.088)	0.39 (0.25, 0.8)	-3.497 (-4.16, -2.83)	11.74 (8.52, 18.59)	-3.134 (-3.702, -2.562)
Turkey	46.9 (41.19, 52.53)	0.381 (0.293, 0.469)	1.55 (1.21, 1.92)	4.024 (3.075, 4.981)	26.17 (21.17, 31.59)	1.123 (0.555, 1.694)
Qatar	44.2 (38.78, 49.5)	-0.463 (-0.834, -0.09)	0.99 (0.7, 1.4)	0.68 (0.325, 1.037)	15.29 (11.61, 19.83)	-0.01 (-0.277, 0.257)
Syria	47.73 (41.42, 54.79)	0.185 (0.123, 0.246)	5.26 (3.85, 9.04)	-3.459 (-4.118, -2.797)	98.5 (74.48, 145.21)	-3.882 (-4.573, -3.187)
Bahrain	45.52 (39.58, 51.59)	-0.009 (-0.048, 0.031)	1.58 (0.62, 4.43)	2.697 (1.831, 3.571)	21.73 (11.28, 51.06)	1.438 (0.802, 2.078)
Jordan	49.19 (43.7, 54.88)	0.462 (0.39, 0.534)	1.41 (1.03, 1.74)	1.761 (1.435, 2.088)	23.92 (18.28, 28.82)	0.975 (0.755, 1.195)
Libya	45.81 (40.07, 52)	0.09 (0.071, 0.11)	0.59 (0.37, 1.43)	1.812 (1.474, 2.15)	14.02 (10.18, 26.28)	1.121 (0.872, 1.371)
Iran	45.73 (40.24, 50.62)	0.072 (0.046, 0.098)	0.81 (0.45, 0.94)	1.262 (0.745, 1.781)	15.72 (9.38, 18.27)	0.871 (0.43, 1.313)
Cape Verde	42.85 (37.33, 48.53)	-0.071 (-0.09, -0.052)	1.06 (0.75, 1.53)	0.771 (0.483, 1.061)	24.4 (19.3, 32.18)	-0.372 (-0.598, -0.146)
Algeria	44.9 (38.64, 50.38)	0.088 (0.066, 0.109)	0.62 (0.41, 1.67)	0.523 (0.256, 0.79)	13.15 (9.8, 26.17)	-0.118 (-0.312, 0.076)
Iraq	43.28 (37.19, 49.38)	0.086 (0.059, 0.114)	0.67 (0.49, 1.2)	-2.773 (-3.158, -2.386)	16.16 (12.47, 21.61)	-3.012 (-3.414, -2.609)
Morocco	42.95 (37.35, 48.88)	0.078 (0.066, 0.091)	0.7 (0.44, 1.81)	1.865 (1.655, 2.075)	15.52 (10.97, 31.32)	1.03 (0.884, 1.175)
Egypt	43.35 (37.36, 49.62)	0.088 (0.074, 0.101)	0.3 (0.18, 0.54)	-0.12 (-0.226, -0.014)	9.21 (6.82, 12.07)	-0.5 (-0.574, -0.426)
Serbia	41.03 (36.82, 45.78)	-0.078 (-0.098, -0.058)	0.74 (0.57, 0.99)	-2.64 (-2.983, -2.295)	16.41 (12.88, 20.99)	-2.859 (-3.18, -2.537)

Saudi Arabia	42.41 (36.46, 48.05)	0.114 (0.084, 0.143)	4.97 (1.91, 6.47)	-0.091 (-0.534, 0.354)	66.76 (32.12, 84.27)	-0.112 (-0.532, 0.311)
Oman	40.78 (35.9, 46.64)	-0.026 (-0.133, 0.08)	1.94 (1.55, 2.37)	3.76 (2.833, 4.696)	33.34 (26.33, 39.67)	2.587 (1.779, 3.401)
Sao Tome and Principe	41.09 (35.23, 46.33)	0.037 (0.021, 0.053)	3.89 (2.37, 5.68)	0.511 (0.263, 0.76)	89.34 (57.59, 126.04)	0.014 (-0.267, 0.295)
Sudan	41.09 (35.69, 46.86)	0.073 (0.049, 0.096)	0.55 (0.35, 1.37)	0.98 (0.694, 1.267)	14.17 (10.01, 26.1)	0.277 (0.046, 0.508)
United Arab Emirates	40.19 (34.97, 45.32)	-0.058 (-0.144, 0.029)	0.7 (0.4, 2.05)	0.044 (-0.542, 0.634)	15.66 (10.72, 33.67)	0.048 (-0.324, 0.422)
Yemen	40.8 (35.72, 46.89)	0.1 (0.082, 0.118)	0.5 (0.31, 1.27)	1.274 (1.007, 1.542)	12.93 (8.98, 24.45)	0.714 (0.502, 0.928)
Mauritania	40.7 (35.13, 46.3)	0.111 (0.095, 0.126)	2.04 (1.45, 3.1)	-1.869 (-1.941, -1.796)	45.89 (31.87, 69.44)	-2.015 (-2.074, -1.956)
Afghanistan	38.95 (33.39, 44.72)	0.001 (-0.028, 0.029)	0.96 (0.56, 2.49)	0.952 (0.825, 1.079)	23.58 (16.14, 47.6)	0.382 (0.257, 0.507)
Senegal	39.47 (33.92, 44.76)	0.05 (0.045, 0.055)	2.39 (1.59, 3.63)	-1.103 (-1.217, -0.989)	56.24 (38, 83.35)	-1.276 (-1.447, -1.104)
Benin	39.29 (33.36, 44.63)	0.036 (0.028, 0.044)	2.21 (1.61, 3.35)	-1.03 (-1.122, -0.938)	56.36 (40.71, 82.97)	-1.103 (-1.177, -1.029)
Ghana	40.2 (34.93, 45.77)	0.133 (0.117, 0.15)	1.95 (1.42, 3.64)	0.373 (0.111, 0.635)	46.43 (33.63, 83.28)	0.231 (-0.019, 0.481)
Togo	39.51 (32.75, 45.67)	0.109 (0.096, 0.123)	2.11 (1.53, 3.06)	-1.2 (-1.342, -1.058)	50.69 (37.25, 72.97)	-1.334 (-1.472, -1.196)
Burkina Faso	39.11 (33.22, 44.42)	0.069 (0.057, 0.08)	3.72 (2.64, 5.09)	-0.172 (-0.357, 0.014)	98.62 (70.21, 134.19)	-0.007 (-0.214, 0.201)
Cameroon	38.76 (33.19, 44.52)	0.004 (-0.005, 0.013)	2.7 (1.91, 4.16)	-1.115 (-1.254, -0.976)	66.25 (45.53, 98.69)	-1.027 (-1.16, -0.894)
Mali	38.75 (33.15, 44.22)	0.043 (0.03, 0.055)	2.22 (1.59, 3.29)	-1.148 (-1.322, -0.974)	59.68 (42.52, 86.59)	-1.285 (-1.489, -1.082)
Chad	37.63 (31.74, 42.8)	-0.072 (-0.106, -0.039)	1.86 (1.38, 2.68)	-1.115 (-1.303, -0.927)	49.05 (36.63, 70.15)	-1.122 (-1.297, -0.947)
Guinea	38.69 (33.38, 44.11)	0.068 (0.057, 0.079)	2.47 (1.76, 3.53)	-0.853 (-0.933, -0.772)	65.9 (48.29, 92.38)	-0.938 (-1.026, -0.849)
Nigeria	41.09 (35.76, 45.67)	0.267 (0.244, 0.29)	1.96 (1.41, 2.86)	-1.084 (-1.211, -0.957)	47.57 (34.81, 67.91)	-1.196 (-1.321, -1.071)
Gambia	39.28 (33.89, 44.19)	0.115 (0.098, 0.132)	2.42 (1.74, 3.61)	-0.044 (-0.171, 0.083)	55.24 (39.12, 81.32)	-0.194 (-0.378, -0.01)
Liberia	38.39 (32.57, 43.71)	0.06 (0.04, 0.08)	2.31 (1.61, 3.44)	-1.447 (-1.673, -1.22)	54.89 (38.86, 79.27)	-1.93 (-2.243, -1.617)
Sierra Leone	37.92 (32.75, 43.78)	-0.009 (-0.026, 0.008)	2.21 (1.59, 3.19)	-0.575 (-0.669, -0.481)	61.78 (43.42, 88.59)	-0.628 (-0.718, -0.539)
Guinea-Bissau	38.42 (32.99, 44.1)	0.054 (0.029, 0.078)	2.42 (1.76, 3.48)	-1.595 (-1.707, -1.482)	61.72 (46.25, 86.35)	-1.789 (-1.894, -1.685)
Niger	38.77 (33.57, 44.56)	0.124 (0.113, 0.135)	1.9 (1.36, 2.84)	-1.537 (-1.671, -1.402)	49.12 (35.64, 70.76)	-1.925 (-2.1, -1.749)
Cote d'Ivoire	37.45 (32.18, 42.3)	0.058 (0.049, 0.068)	2.17 (1.57, 3.19)	-1.208 (-1.351, -1.064)	52.02 (36.78, 75.08)	-1.297 (-1.447, -1.147)
Comoros	36.21 (31.73, 40.94)	0.063 (0.054, 0.072)	2.68 (1.34, 4.44)	0.089 (-0.035, 0.213)	60.23 (30.88, 98.94)	-0.17 (-0.388, 0.049)
Kenya	35.96 (31.53, 40.23)	0.149 (0.107, 0.192)	2.76 (1.53, 4.43)	0.966 (0.823, 1.109)	60.57 (35.29, 93.93)	0.955 (0.762, 1.148)

Tanzania	35.64 (30.98, 40.35)	0.041 (0.024, 0.057)	2.3 (1.07, 3.92)	0.632 (0.486, 0.778)	54.71 (27.79, 90.38)	0.49 (0.336, 0.645)
Mozambique	35.49 (30.37, 40.59)	0.075 (0.063, 0.088)	3.49 (1.71, 5.93)	0.853 (0.755, 0.952)	81.18 (42.42, 137.28)	0.836 (0.701, 0.971)
Rwanda	35.94 (31.16, 40.99)	0.138 (0.119, 0.158)	2.9 (1.33, 4.96)	-0.598 (-0.77, -0.426)	64.79 (31.32, 107.77)	-1.214 (-1.424, -1.004)
Madagascar	35.03 (30.03, 39.56)	0.018 (0.004, 0.033)	1.98 (1.09, 3.23)	-0.103 (-0.229, 0.023)	47.24 (27.19, 76.22)	-0.39 (-0.455, -0.324)
Gabon	35.96 (30.51, 41.55)	0.167 (0.156, 0.178)	3.27 (1.91, 5.41)	0.232 (0.076, 0.389)	67.23 (40.48, 109.26)	-0.141 (-0.304, 0.022)
Burundi	34.2 (29.38, 39.01)	-0.049 (-0.059, -0.04)	2.68 (1.37, 4.51)	-0.143 (-0.307, 0.021)	62.83 (34.56, 101.26)	-0.514 (-0.656, -0.371)
Djibouti	34.96 (29.78, 39.4)	0.095 (0.076, 0.114)	2.98 (1.34, 5.23)	0.975 (0.731, 1.22)	66.34 (30.49, 116.36)	0.7 (0.433, 0.967)
Equatorial Guinea	34.66 (29.58, 39.62)	0.103 (0.056, 0.15)	2.75 (1.52, 4.53)	-0.341 (-0.685, 0.004)	54.52 (30.51, 89.65)	-1.198 (-1.565, -0.83)
Zambia	35.03 (30.03, 40.26)	0.08 (0.069, 0.092)	3.04 (1.53, 5)	-0.12 (-0.331, 0.091)	70.51 (38.1, 112.46)	-0.391 (-0.613, -0.168)
Congo	34.79 (29.64, 39.79)	0.075 (0.061, 0.088)	2.89 (1.6, 4.76)	-0.824 (-1.031, -0.617)	61.44 (35.97, 97.79)	-1.207 (-1.419, -0.994)
Uganda	35.4 (29.98, 40.68)	0.119 (0.109, 0.128)	2.33 (1.22, 3.79)	0.337 (0.136, 0.538)	52.76 (29.61, 85.08)	0.184 (-0.063, 0.431)
Eritrea	34.59 (29.52, 39.21)	0.072 (0.061, 0.083)	2.65 (1.45, 4.28)	0.417 (0.232, 0.602)	63.61 (35.71, 102.21)	0.081 (-0.094, 0.257)
Ethiopia	34.83 (30.35, 38.88)	0.119 (0.096, 0.141)	2.81 (1.42, 4.99)	-2.302 (-2.451, -2.153)	59.89 (32.41, 102.83)	-3.004 (-3.143, -2.865)
Malawi	35.12 (29.69, 39.77)	0.133 (0.122, 0.145)	2.61 (1.39, 4.37)	-0.125 (-0.213, -0.037)	60.63 (33.76, 102.65)	-0.304 (-0.411, -0.196)
South Sudan	35.43 (30.79, 40.25)	0.208 (0.189, 0.226)	2.19 (1.16, 3.77)	0.024 (-0.009, 0.056)	49.49 (28.22, 82.35)	-0.257 (-0.311, -0.202)
DR Congo	34.12 (29.43, 38.99)	0.039 (0.024, 0.054)	2.52 (1.35, 4.25)	-0.293 (-0.357, -0.229)	56.64 (31.96, 95)	-0.36 (-0.431, -0.288)
Somalia	34.25 (29.51, 38.98)	0.038 (0.029, 0.048)	3.41 (1.69, 5.87)	0.548 (0.451, 0.645)	84.14 (44.42, 142.48)	0.402 (0.312, 0.492)
Central African Republic	33.13 (28.22, 38.12)	0.055 (0.032, 0.078)	3.14 (1.66, 5.69)	-0.178 (-0.259, -0.098)	77.51 (43.92, 132.48)	-0.267 (-0.352, -0.181)
Angola	34.89 (29.86, 39.81)	0.217 (0.211, 0.224)	2.56 (1.45, 4.31)	-1.002 (-1.162, -0.842)	57.61 (33.62, 96.31)	-1.319 (-1.475, -1.163)
Vietnam	28.95 (24.91, 32.9)	-0.082 (-0.114, -0.049)	1.37 (0.6, 1.79)	-0.531 (-1.048, -0.011)	28.98 (12.75, 37.76)	-0.624 (-0.993, -0.255)
Malaysia	25.53 (21.82, 29.31)	0.045 (0.016, 0.075)	3.39 (1.68, 8.5)	1.614 (1.325, 1.905)	64.66 (35.07, 145.57)	1.183 (0.906, 1.46)
Thailand	25.4 (22.16, 28.92)	0.053 (0.009, 0.097)	2.53 (1.55, 4.69)	-2.894 (-3.419, -2.366)	49.37 (32.22, 82.04)	-3.674 (-4.246, -3.099)
Seychelles	24.82 (21.45, 27.89)	-0.01 (-0.062, 0.042)	11.03 (5.37, 13.37)	3.206 (2.859, 3.554)	202.05 (111.76, 240.56)	2.717 (2.416, 3.019)
Sri Lanka	26.29 (22.57, 30.13)	0.279 (0.246, 0.312)	3.72 (2.12, 5.08)	3.689 (2.631, 4.759)	79.17 (41.11, 109.93)	3.459 (2.467, 4.461)
Mauritius	25.59 (21.88, 29.11)	0.155 (0.134, 0.176)	2.03 (1.07, 2.6)	4.626 (3.913, 5.342)	43.69 (24.58, 56.34)	4.163 (3.523, 4.806)
Philippines	24.99 (21.92, 27.94)	0.185 (0.131, 0.239)	5.22 (4.29, 6.14)	-0.115 (-0.283, 0.054)	121.92 (97.27, 144.4)	-0.153 (-0.313, 0.008)

Maldives	22.27 (19.25, 25.09)	-0.178 (-0.287, -0.07)	2.19 (1.49, 2.88)	-1.838 (-2.015, -1.661)	37.17 (28, 47.05)	-2.476 (-2.699, -2.252)
Cambodia	23.6 (20.07, 27.49)	-0.01 (-0.046, 0.027)	3.49 (2.59, 4.27)	-0.06 (-0.135, 0.016)	77.24 (56.31, 95.78)	-0.714 (-0.798, -0.629)
Indonesia	19.93 (17.26, 22.38)	-0.727 (-0.902, -0.552)	3.3 (2.66, 3.88)	0.493 (0.418, 0.568)	74.95 (57.2, 88.64)	-0.147 (-0.207, -0.087)
Myanmar	23.84 (20.06, 27.41)	0.133 (0.099, 0.168)	3.44 (2.63, 4.42)	-0.709 (-0.926, -0.491)	79.98 (59.63, 102.57)	-1.353 (-1.612, -1.094)
Timor-Leste	23.03 (20.12, 26.14)	0.088 (0.068, 0.108)	2.87 (2, 3.71)	0.229 (0.057, 0.402)	61.8 (40.38, 80.43)	-0.489 (-0.751, -0.227)
Laos	22.7 (19.61, 25.72)	0.06 (0.037, 0.083)	3.05 (2.17, 3.93)	-0.938 (-1.11, -0.765)	70.2 (48.4, 91.15)	-1.543 (-1.722, -1.364)
Guam	21.37 (18.31, 24.2)	0.064 (0.004, 0.124)	3.16 (2.48, 3.88)	-2.626 (-3.279, -1.968)	63.08 (50.88, 76.68)	-1.657 (-2.163, -1.149)
Tonga	20.85 (17.94, 23.82)	0.036 (0.007, 0.065)	3.98 (3.09, 5.06)	-0.204 (-0.489, 0.081)	78.21 (59.97, 100.22)	-0.159 (-0.433, 0.117)
American Samoa	20.8 (17.92, 23.64)	0.089 (0.056, 0.122)	7.47 (5.15, 9.04)	-0.853 (-1.226, -0.478)	135.36 (102.82, 162.77)	-0.854 (-1.217, -0.49)
Tuvalu	18.58 (16.05, 21.53)	-0.28 (-0.299, -0.261)	3.48 (2.65, 4.67)	-1.378 (-1.485, -1.271)	75.18 (56.41, 100.89)	-1.638 (-1.706, -1.57)
Niue	20.33 (17.53, 23.13)	-0.002 (-0.018, 0.015)	2.89 (1.89, 3.76)	-1.352 (-1.488, -1.216)	60.01 (40.02, 79.37)	-1.376 (-1.519, -1.233)
Cook Islands	21.49 (18.62, 24.61)	0.286 (0.27, 0.302)	0.3 (0.22, 0.46)	-0.772 (-1.02, -0.523)	10.4 (7.38, 13.62)	-0.569 (-0.773, -0.364)
Tokelau	19.52 (16.63, 22.1)	-0.121 (-0.159, -0.082)	2.86 (2.21, 3.63)	-1.28 (-1.335, -1.226)	60.28 (44.71, 80.62)	-1.481 (-1.57, -1.392)
Palau	18.47 (15.6, 21.22)	-0.152 (-0.226, -0.077)	2.78 (1.67, 3.75)	-0.8 (-0.86, -0.741)	58.15 (34.51, 79.33)	-0.735 (-0.769, -0.7)
Fiji	19.36 (16.33, 22.12)	-0.007 (-0.023, 0.009)	2.64 (1.67, 3.36)	1.554 (1.319, 1.789)	49.42 (33.91, 62.49)	1.429 (1.174, 1.684)
Northern Mariana Islands	20.48 (17.71, 23.23)	0.09 (-0.016, 0.195)	6.37 (4.74, 7.74)	-1.598 (-1.871, -1.325)	111.77 (87.05, 137)	-1.607 (-1.88, -1.333)
Samoa	19.46 (16.84, 22.06)	0.033 (0.002, 0.064)	3.37 (2.62, 4.21)	-1.388 (-1.498, -1.277)	69.83 (52.81, 88.88)	-1.293 (-1.381, -1.205)
Kiribati	19.17 (16.28, 22.05)	0.08 (0.051, 0.11)	6.32 (4.81, 8.06)	-0.379 (-0.435, -0.323)	133.63 (101.6, 172.9)	-0.756 (-0.816, -0.696)
Micronesia	18.96 (16.22, 21.53)	0.056 (0.012, 0.1)	5 (3.83, 6.61)	-0.427 (-0.582, -0.271)	106.73 (76.8, 140.96)	-0.554 (-0.712, -0.395)
Marshall Islands	18.87 (15.75, 21.92)	0.066 (0.042, 0.091)	4.34 (3.21, 6.42)	-0.372 (-0.462, -0.282)	97.73 (71.97, 138.45)	-0.255 (-0.389, -0.121)
Nauru	19.43 (16.64, 22.71)	0.197 (0.182, 0.212)	4.22 (3.2, 5.19)	-0.767 (-1.052, -0.482)	95.81 (68.71, 121.41)	-0.731 (-1.095, -0.367)
Solomon Islands	18.75 (16.05, 21.66)	0.12 (0.103, 0.138)	4.08 (3.08, 5.21)	-0.187 (-0.29, -0.083)	105.63 (68.2, 141.47)	-0.175 (-0.306, -0.044)
Vanuatu	18.31 (15.71, 21.2)	0.053 (0.037, 0.068)	3.64 (2.68, 5.26)	-0.207 (-0.308, -0.105)	82.03 (58.98, 116.48)	-0.109 (-0.233, 0.016)
Papua New Guinea	17.93 (15.27, 20.63)	-0.009 (-0.023, 0.005)	2.43 (1.78, 3.58)	-0.465 (-0.497, -0.432)	58.15 (43.13, 79.31)	-0.326 (-0.388, -0.265)
Taiwan (Province of China)	14.31 (12.17, 16.25)	0.204 (0.174, 0.234)	6.88 (4.24, 8.76)	4.499 (3.333, 5.678)	94.45 (60.08, 121.82)	3.868 (2.761, 4.987)

North Korea	12.11 (10.57, 13.81)	-0.24 (-0.252, -0.228)	1.05 (0.82, 1.39)	-1.08 (-1.234, -0.925)	27.12 (20.37, 36.01)	-1.32 (-1.422, -1.218)
China	12.28 (10.7, 13.78)	-0.202 (-0.277, -0.126)	0.44 (0.35, 0.64)	-2.748 (-3.176, -2.317)	9.95 (8.23, 13.33)	-3.185 (-3.62, -2.749)

ASIR, age-standardized incidence year rate; ASMR, age-standardized mortality year rate; ASDR, age-standardized disability-adjusted life year rate; UI, uncertainty interval; EAPC, estimated annual percentage change; CI, confidential interval.

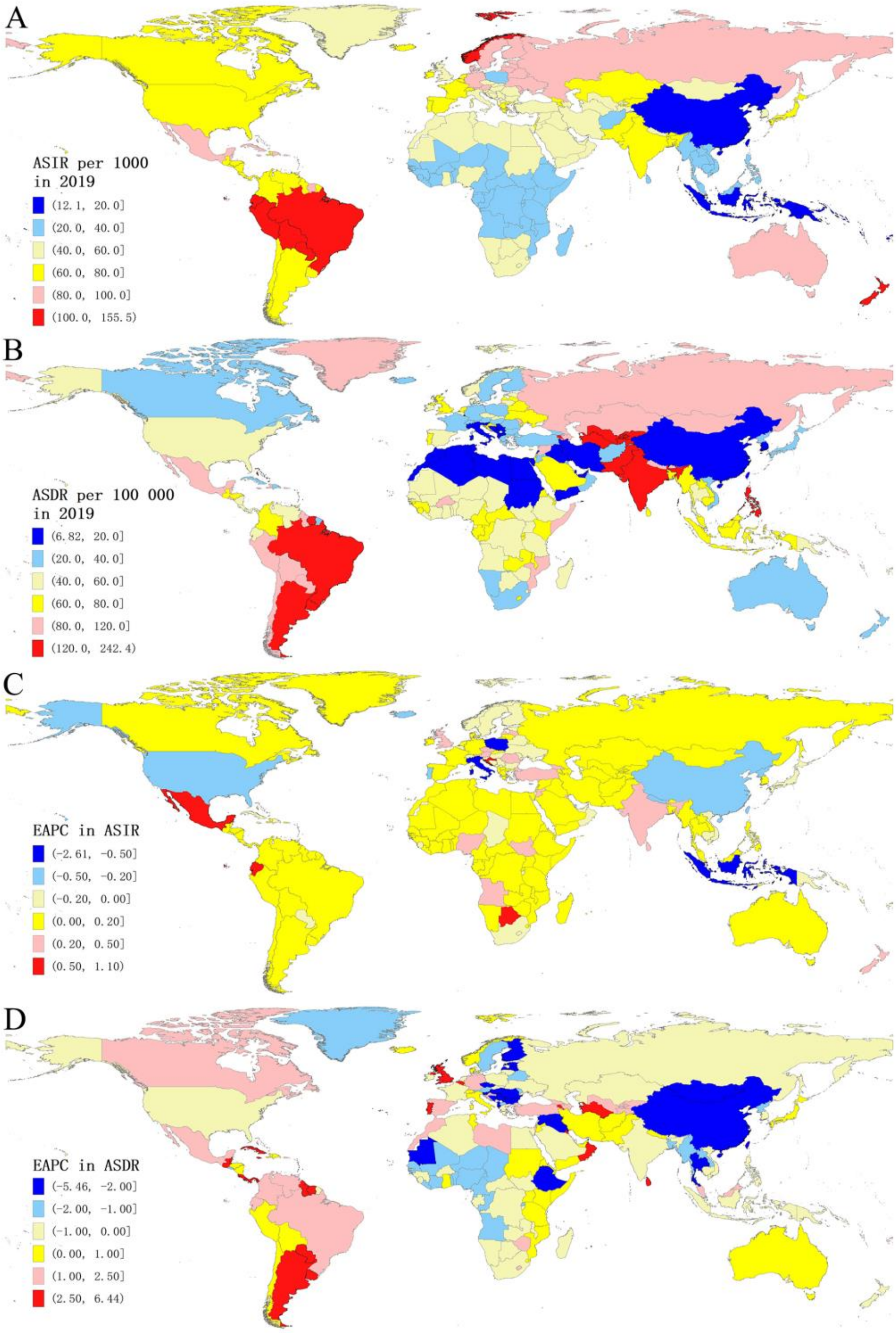

Figure S1. Global incidence and DALYs of urinary tract infection for both sexes across 204 countries and territories. (A) ASIR of urinary tract infection in 2019; (B) ASDR of urinary tract infection in 2019; (C) EAPC in the ASIR of urinary tract infection from 1990 to 2019; (D) EAPC in the ASDR associated with urinary tract infection from 1990 to 2019. DALYs, disability-adjusted life years; ASIR, age-standardized incidence rate; ASDR, age-standardized DALYs rate; EAPC, estimated annual percentage change

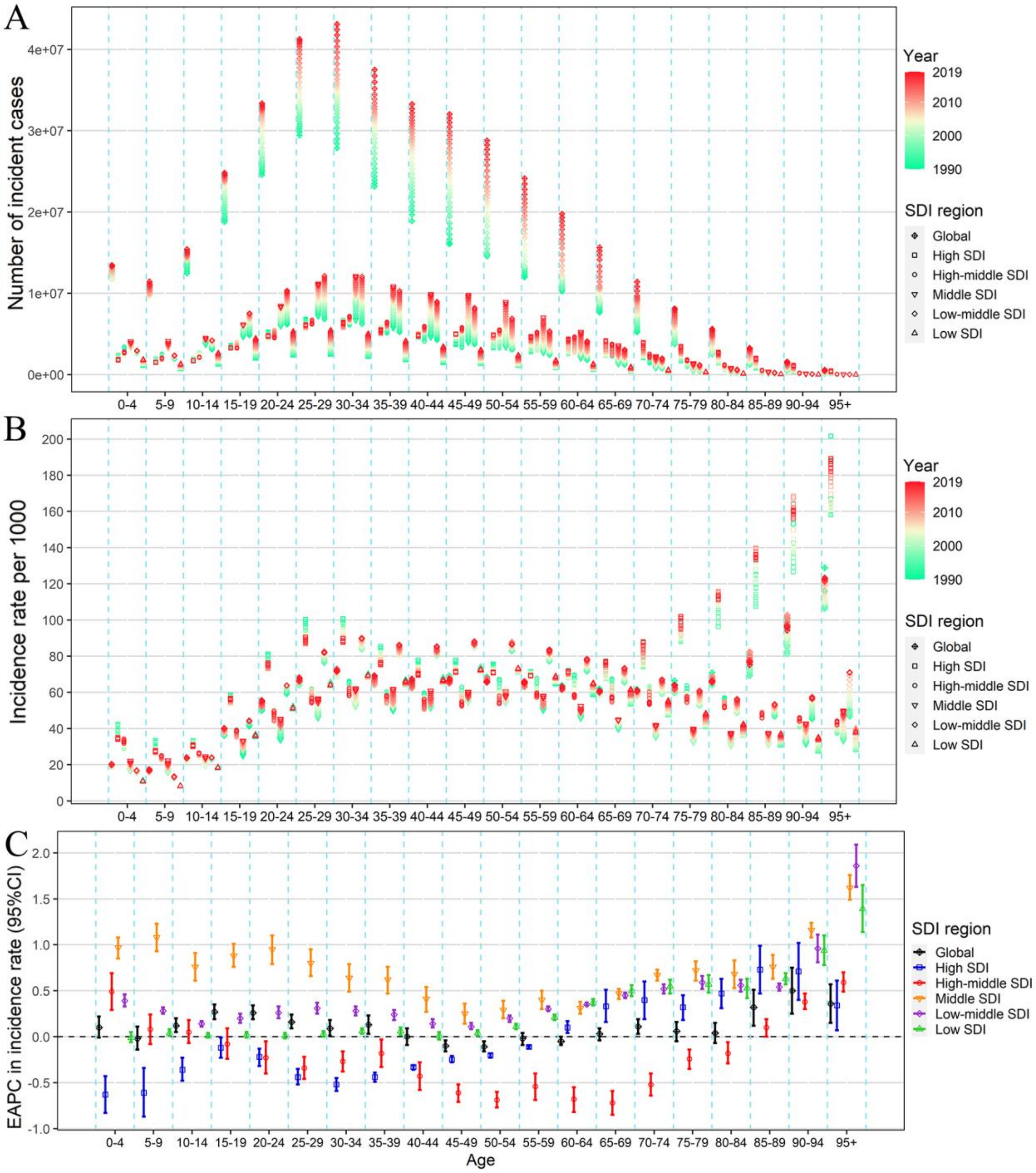

Figure S2. Change in the incidence of urinary tract infection across all age groups in the worldwide and five SDI regions, both sexes, from 1990 to 2019. (A) Number of cases; (B) Age-specific incidence rate; (C) EAPC in the age-specific incidence rate of urinary tract infection; EAPC, estimated annual percentage change.

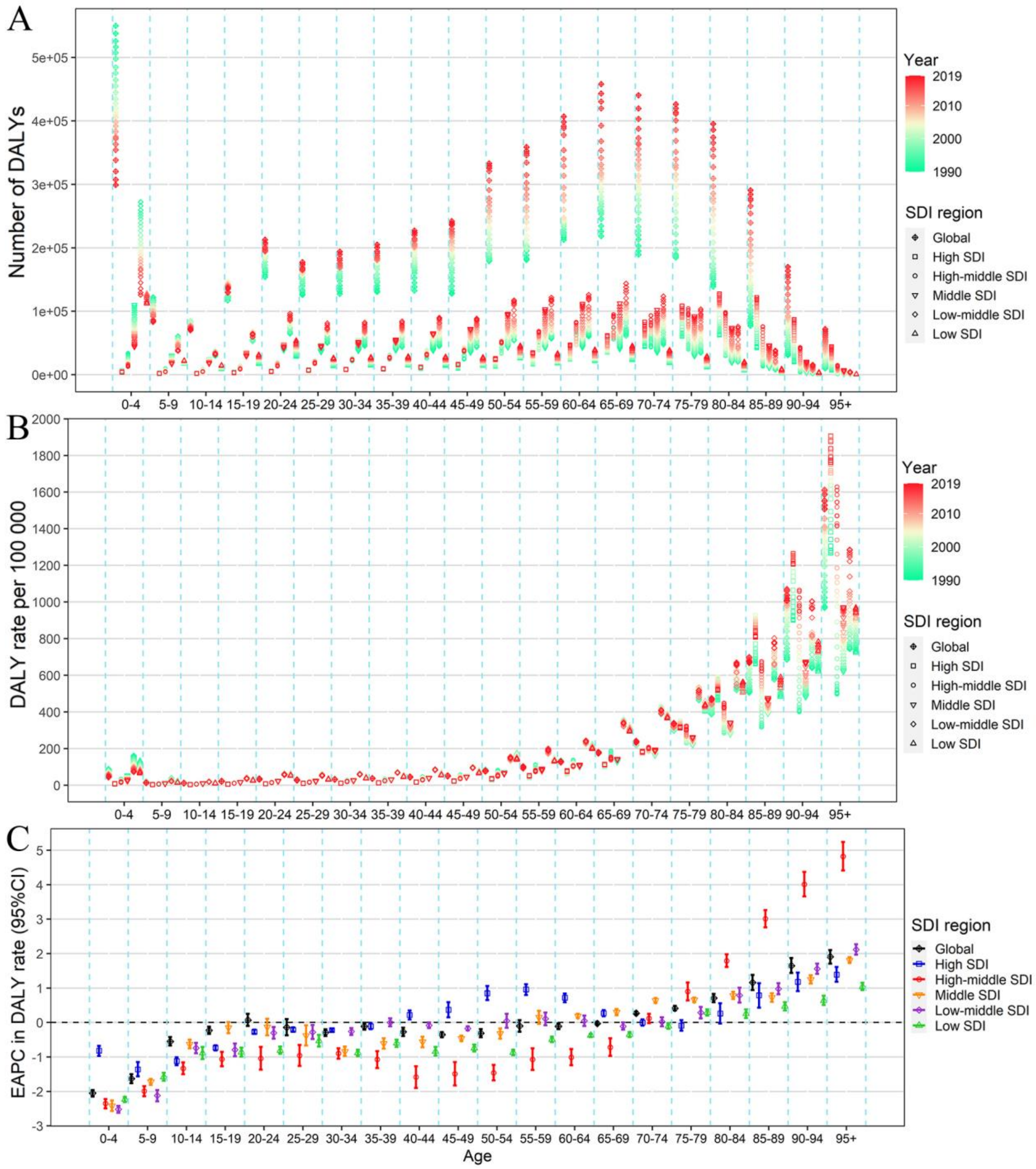

Figure S3. Changes in DALYs associated with urinary tract infection across all age groups in the worldwide and five SDI regions, both sexes, from 1990 to 2019. (A) Number of cases of urinary tract infection; (B) Age-specific incidence rate of urinary tract infection; (C) EAPC in the age-specific incidence rate of urinary tract infection; DALYs, disability-adjusted life years; EAPC, estimated annual percentage change.

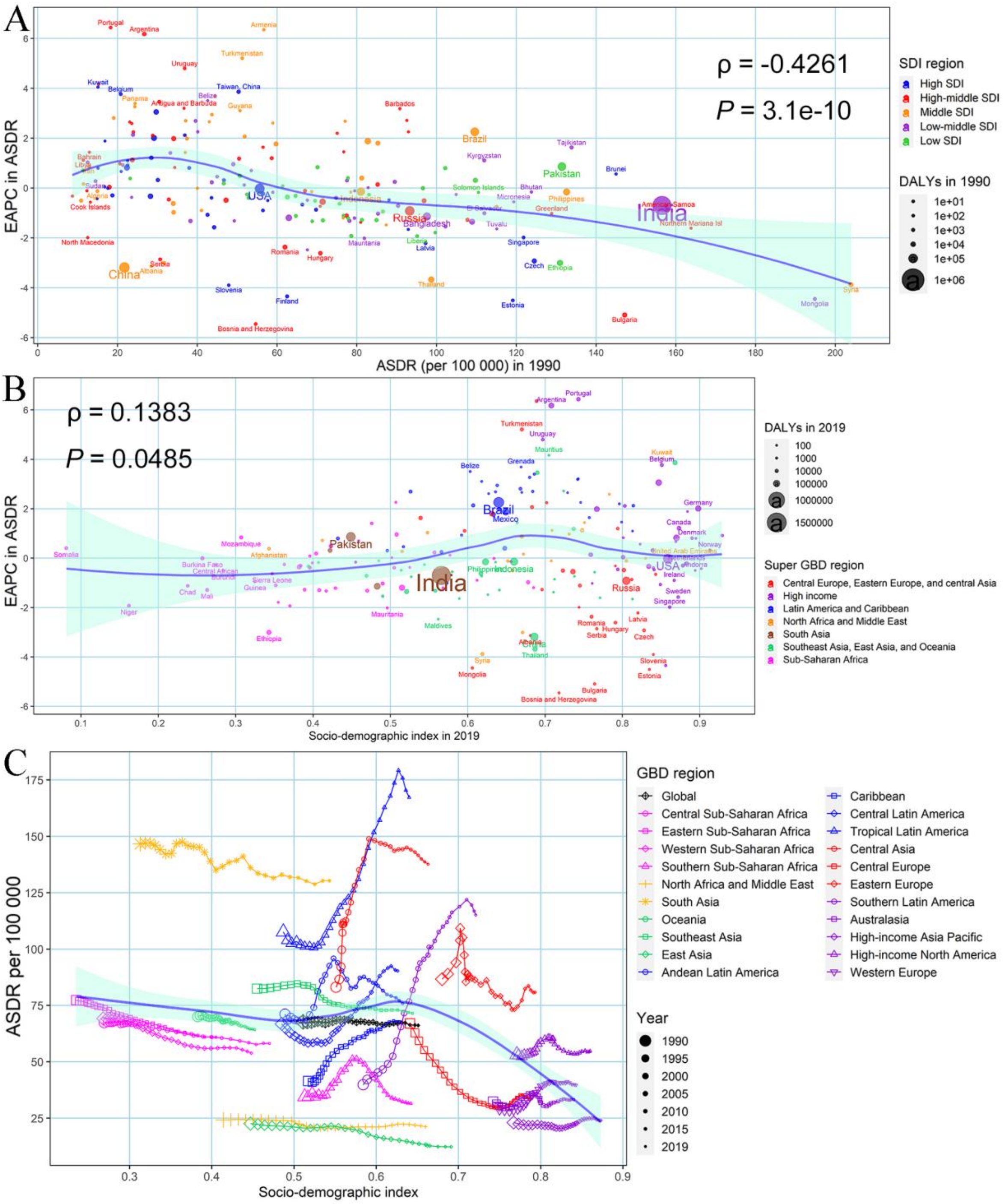

Figure S4. Factors associated the EAPC in ASDR associated with urinary tract infection in both sexes from 1990 to 2019. (A) ASDR associated with urinary tract infection in 1990 at the national and territorial level; (B) SDI associated with urinary tract infection in 2019 at the national and territorial level; (C) Annual change in ASDR associated with urinary tract infection across 21 GBD regions according to SDI. The blue line was fitted by LOESS. ASDR, age-standardized disability-adjusted life year rate; EAPC, estimated annual percentage change; SDI, socio-demographic index

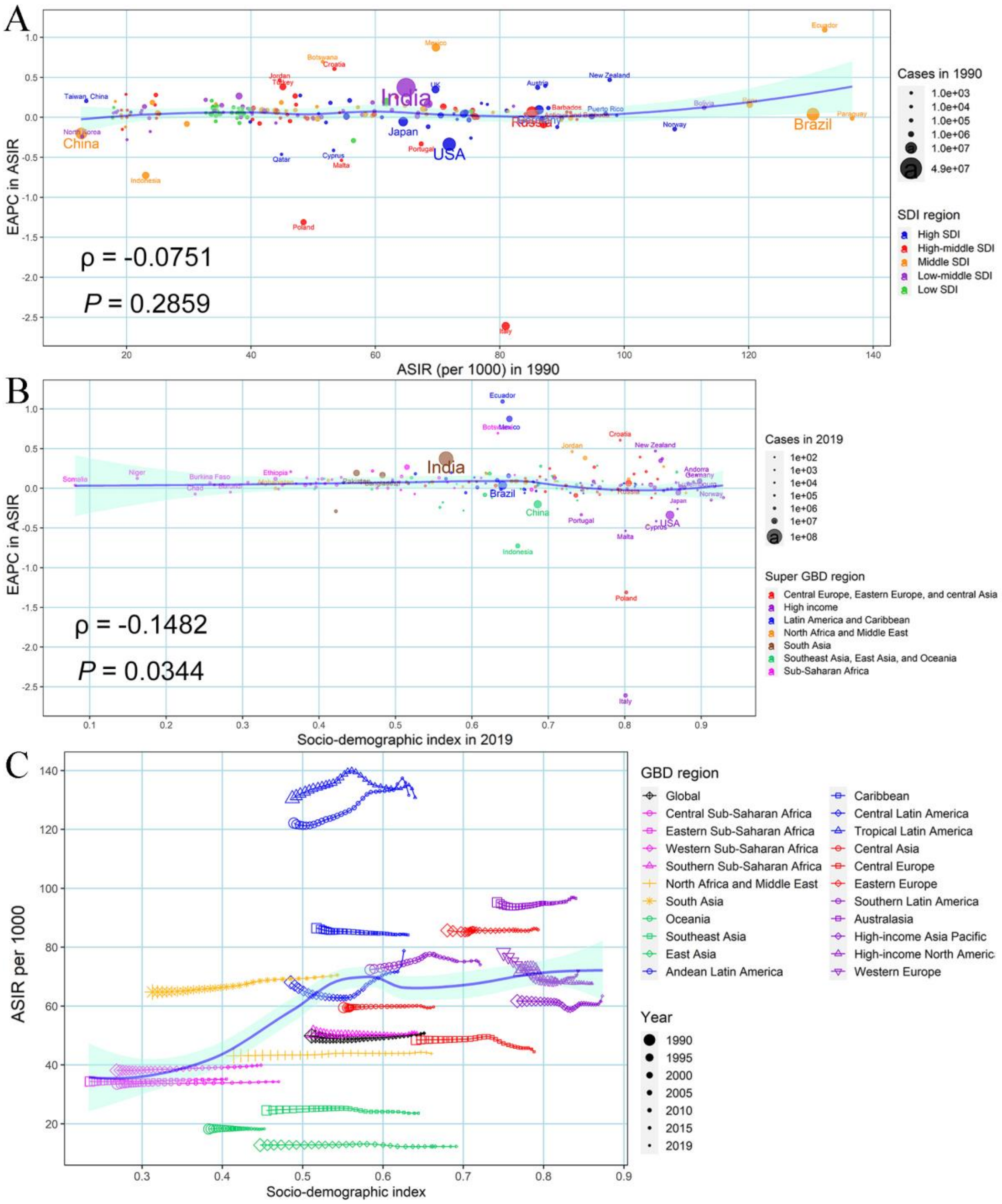

Figure S5. Factors associated with EAPC in the ASIR of urinary tract infection in both sexes from 1990 to 2019. (A) ASIR of urinary tract infection in 1990 at the national and territorial level; (B) SDI associated with urinary tract infection in 2019 at the national and territorial level; (C) Annual change in the ASIR of urinary tract infection across 21 GBD regions according to SDI. The blue line was fitted by LOESS. ASIR, age-standardized incidence rate; EAPC, estimated annual percentage change; SDI, socio-demographic index.
